# Vincristine Beyond Mitosis: Uncovering a First Link to G-Quadruplex DNA in Cancer Cells

**DOI:** 10.3390/ijms26199606

**Published:** 2025-10-01

**Authors:** Anna Di Porzio, Carolina Persico, Francesca Romano, Alessandra Barra, Immacolata Aiello, Ludovica D’Auria, Sara Abate, Federica D’Aria, Concetta Giancola, Elpidio Cinquegrana, Francesco Saverio Di Leva, Jussara Amato, Simona Marzano, Nunzia Iaccarino, Antonio Randazzo

**Affiliations:** 1 Department of Pharmacy, University of Naples Federico II, Via D. Montesano 49, 80131 Naples, Italy; anna.diporzio@unina.it (A.D.P.); carolina.persico@unina.it (C.P.); francesca.romano2@unina.it (F.R.); aless.barra@studenti.unina.it (A.B.); imma.aiello@studenti.unina.it (I.A.); sar.abate@studenti.unina.it (S.A.); federica.daria@unina.it (F.D.); giancola@unina.it (C.G.); elpidio.cinquegrana@unina.it (E.C.); francesco.dileva@unina.it (F.S.D.L.); jussara.amato@unina.it (J.A.); simona.marzano@unina.it (S.M.); 2 CEINGE—Biotecnologie Avanzate Franco Salvatore, 80145 Naples, Italy; daurial@ceinge.unina.it

**Keywords:** vincristine, G-quadruplex DNA, immunofluorescence, circular dichroism, nuclear magnetic resonance, isothermal titration calorimetry, U2OS cells, HeLa cells, MDA-MB-231 cells, mitochondria

## Abstract

Vincristine is a classical chemotherapeutic agent widely used for its ability to disrupt microtubule polymerization, yet additional molecular effects may contribute to its anticancer activity. G-quadruplexes (G4s), non-canonical nucleic acid structures enriched in regulatory regions of the genome and in mitochondrial DNA, have emerged as relevant modulators of cellular homeostasis. In this study, we investigated whether vincristine can influence G4 biology. Cancer cells treated with vincristine were analyzed by immunofluorescence, revealing a consistent increase in nuclear and mitochondrial G4 foci. In particular, mitochondrial G4s were significantly elevated by approximately 1.5–2.5 fold compared to untreated cells, an effect accompanied by a detectable reduction in membrane potential, indicative of impaired organelle function. In addition, biophysical analyses on representative G4-forming sequences were carried out. Proton nuclear magnetic resonance titrations showed localized chemical shift perturbations upon vincristine addition, circular dichroism confirmed preservation of G4 topology, and isothermal titration calorimetry indicated weak but enthalpically favorable interactions. Taken together, these results suggest that vincristine perturbs both the cellular G4 landscape and mitochondrial homeostasis, while also engaging G4 DNA in vitro. Although additional studies are required to establish the mechanistic details, this work provides proof-of-concept for a previously unrecognized dimension of vincristine’s anticancer action.

## 1. Introduction

Cancer remains a leading cause of mortality worldwide, representing a persistent challenge due to its remarkable molecular heterogeneity and complexity [1]. Hallmark features such as genomic instability, epigenetic dysregulation, metabolic rewiring, and extensive cellular plasticity drive tumor progression and contribute to therapeutic failure [2,3]. In this context, the discovery of novel molecular targets is essential to overcoming resistance and improving treatment efficacy.

**Figure 1:**
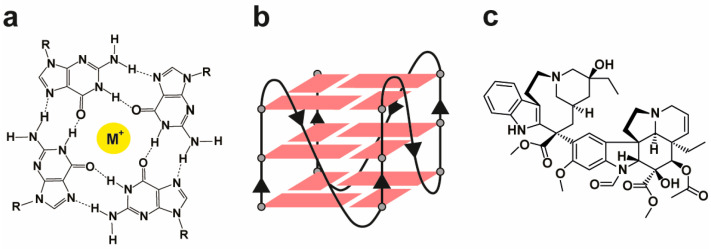
(**a**) Chemical depiction of a G-tetrad stabilized by Hoogsteen hydrogen bonds and a central monovalent cation (M^+^). (**b**) Schematic illustration of a G-quadruplex structure formed by the stacking of G-tetrads. (**c**) Molecular structure of vincristine.

Among emerging targets, non-canonical nucleic acid structures have garnered growing attention for their regulatory potential in cancer biology. A prominent example is the G-quadruplex (G4): a four-stranded secondary DNA (or RNA) structure formed by stacked guanine tetrads stabilized by Hoogsteen hydrogen bonding and monovalent cations (e.g., K^+^ or Na^+^) [4,5] (Figure 1a,b). Genome-wide analyses have revealed over 700,000 putative G4-forming sequences (PQSs) within the human genome, particularly enriched in functionally critical regions such as oncogene promoters, telomeres, replication origins, and untranslated regions (UTRs) of mRNAs. This non-random distribution suggests a broad involvement of G4s in key cellular processes, including transcriptional regulation, DNA replication, genome stability, and RNA splicing [6,7,8,9,10].

Thanks to their distinctive structural features and strategic genomic positioning, G4s have emerged as attractive therapeutic targets in oncology. Indeed, a growing number of small molecules, collectively referred to as G4 ligands, have been developed or repurposed to selectively recognize and stabilize G4 structures [11]. These compounds aim to modulate oncogene expression, induce replication stress, or activate DNA damage responses in tumor cells. Crucially, the therapeutic efficacy of G4 ligands depends not only on their affinity and selectivity for G4s over duplex DNA, but also on their potential to act synergistically with established anticancer agents [12].

In a recent study published by our group [13], we systematically investigated the combinatorial effects of three well-characterized G4 ligands—berberine, RHPS4, and pyridostatin—with seven traditional chemotherapeutics in a cancer cell model [14]. We tested both simultaneous and sequential drug administration protocols to identify conditions that maximize synergy. Among all tested combinations, vincristine (Figure 1c), a classical Vinca alkaloid known for its anti-mitotic action via β-tubulin binding and microtubule destabilization [15,16,17,18], stood out for its pronounced synergistic effect when administered 24 h prior to RHPS4.

This unexpected interaction led us to hypothesize that vincristine may recognize G4 DNA structures independently of its canonical mechanism. In this study, we explore this possibility through a comprehensive set of biological and biophysical assays aimed at determining whether vincristine can directly bind to G4 structures. We assess its biological impact on the G4 landscape, evaluate its binding specificity, and characterize its structural preferences. Demonstrating such an interaction would not only redefine our understanding of vincristine’s mechanism of action, but also provide a rationale for its repurposing as potential G4-targeting agent—opening new avenues for rational combination therapies in oncology.

## 2. Results and Discussion

### 2.1. Immunofluorescence

**Figure 2:**
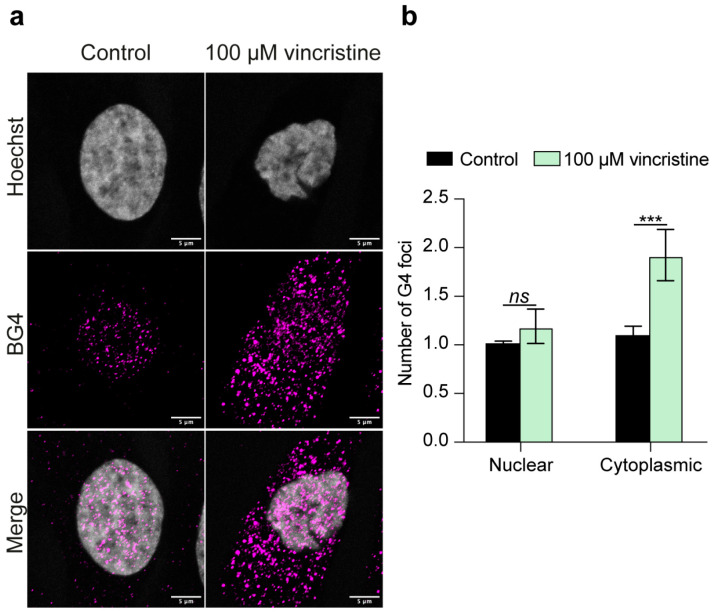
(**a**) Representative immunofluorescence images showing G4 foci formation in HeLa cells treated for 24 h with either 0.5% DMSO (control) or 100 μM vincristine. Nuclei were stained with the Hoechst solution (grey) and G4 structures with BG4 (magenta). Merged channels are also reported. Scale bar: 5 μm. (**b**) Quantitative analysis of nuclear and cytoplasmic G4 foci. Data is shown as the mean ± SD of two independent experiments and is expressed as fold change over DMSO-treated cells (negative control). Approximately 100 cells were screened per condition. The statistical significance was calculated using a Student’s *t*-test on GraphPad Prism 10.2.1 (ns: not significant; ***: *p* < 0.001). Refer to Figure S1 for a larger field image.

As a first step in this study, we aimed to assess the impact of vincristine on the G-quadruplex (G4) landscape in different cancer cell lines, including HeLa (cervical adenocarcinoma) and U2OS (osteosarcoma) cells—which have been mapped in detail for G4 distribution and dynamics [19]—as well as the highly aggressive and metastatic MDA-MB-231 human breast cancer cell line, largely employed for testing promising G4 binders [20]. In particular, cells were treated for 24 h with either vehicle or vincristine, and subjected to immunofluorescence analysis using the highly specific G4-recognizing BG4 antibody [21]. Intriguingly, vincristine treatment elicited different effects on G4 structures in the investigated cell lines. In HeLa cells, the nuclear G4 content remained largely unaffected, whereas the number of cytoplasmic G4 foci nearly doubled (Figure 2).

**Figure 3:**
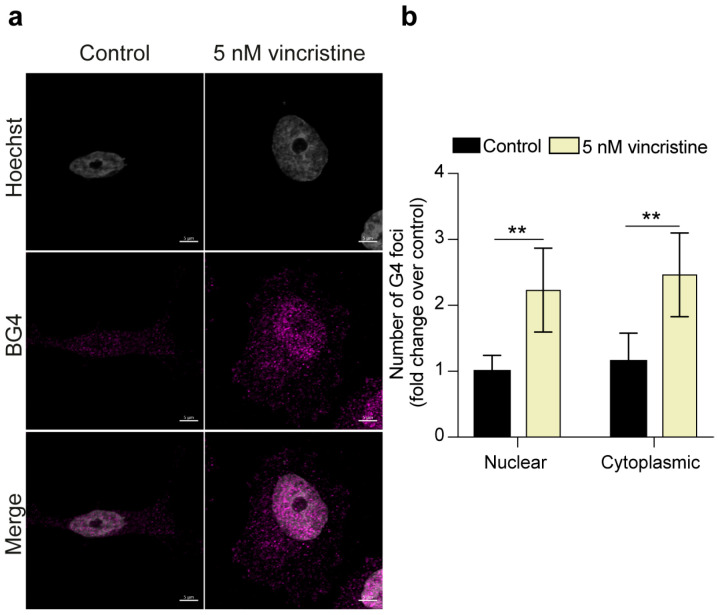
(**a**) Representative immunofluorescence images showing G4 foci formation in MDA-MB-231 cells following a 24 h treatment with either 0.5% DMSO (control) or 5 nM vincristine. Nuclei were stained with the Hoechst solution (grey) and G4 structures with BG4 (magenta). Merged channels are also reported. Scale bar: 5 μm. (**b**) Quantitative analysis of nuclear and cytoplasmic G4 foci. Results, expressed as fold change over DMSO-treated control, represent the mean ± SD of two independent experiments. Approximately 100 cells were screened per condition. The statistical significance was calculated using a Student’s *t*-test on GraphPad Prism 10.2.1 (**: *p* < 0.01). Refer to Figure S2 for a larger field image.

Intriguingly, quantitative analysis of MDA-MB-231 cells revealed that the number of G4 foci was more than doubled in both the nuclear and cytoplasmic cellular compartments after treatment with the chemotherapeutic (Figure 3).

**Figure 4:**
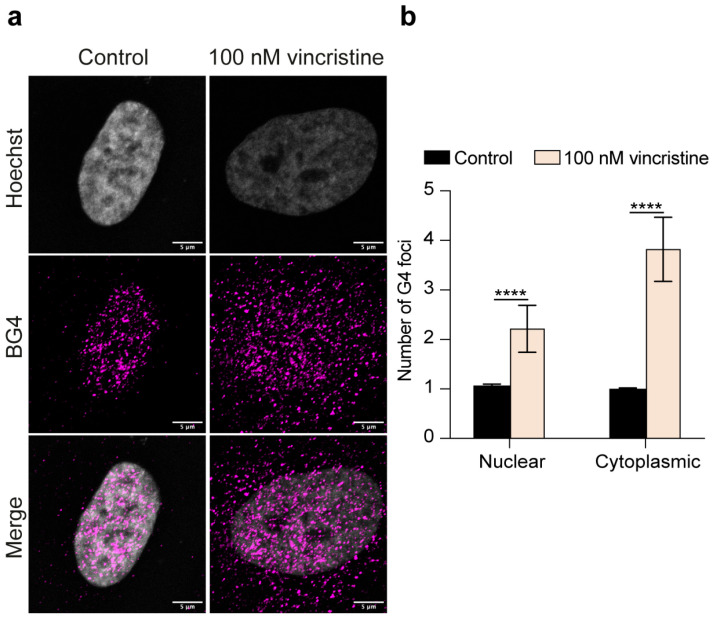
(**a**) Representative immunofluorescence images showing G4 foci formation in U2OS cells following a 24 h treatment with either 0.5% DMSO (control) or 100 nM vincristine. Nuclei were stained with the Hoechst solution (grey) and G4 structures with BG4 (magenta). Merged channels are also reported. Scale bar: 5 μm. (**b**) Quantitative analysis of nuclear and cytoplasmic G4 foci. Results, expressed as fold change over DMSO-treated control, represent the mean ± SD of two independent experiments. Approximately 100 cells were screened per condition. The statistical significance was calculated using a Student’s *t*-test on GraphPad Prism 10.2.1 (****: *p* < 0.0001). Refer to Figure S3 for a larger field image.

Interestingly, U2OS cells displayed an even more widespread response to vincristine, with roughly a two-fold increase in nuclear G-quadruplex structures and a four-fold increase in cytoplasmic G4s following vincristine exposure (Figure 4). These results collectively underscore vincristine’s marked ability to perturb the overall G4 landscape in human cancer cells, suggesting a pivotal role for G-quadruplex structures in mediating cancer cells’ response to the drug.

**Figure 5:**
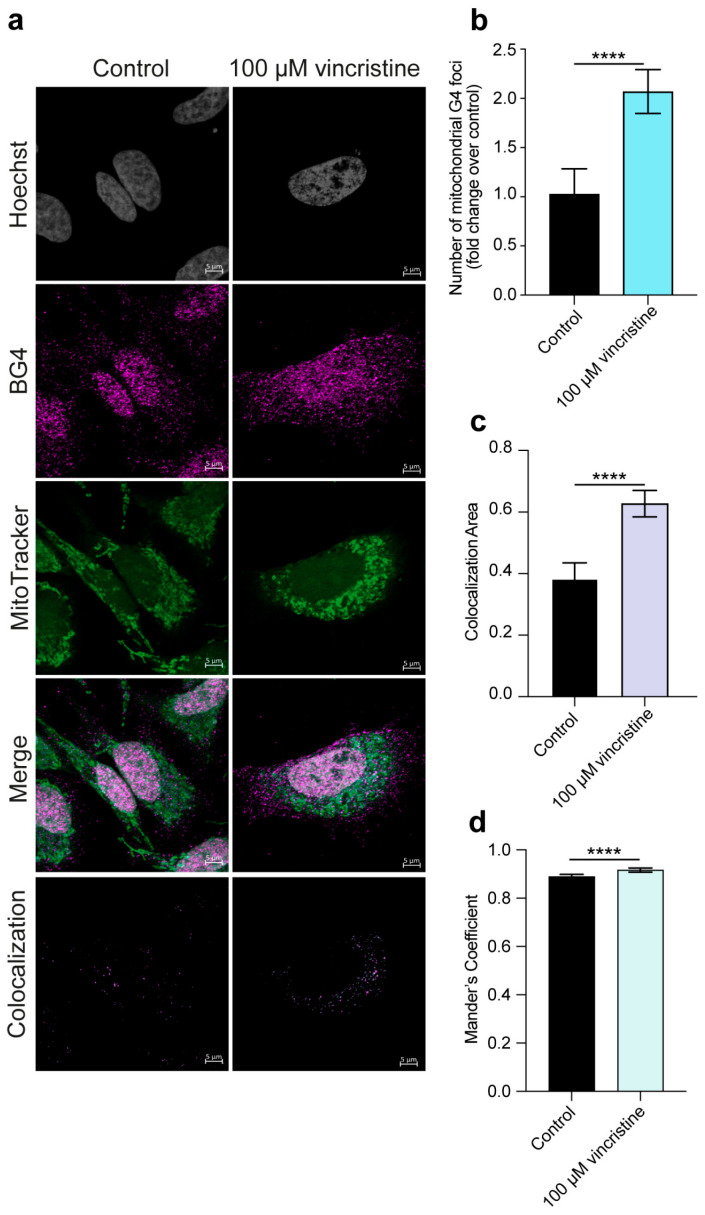
(**a**) Representative immunofluorescence images showing G4 foci formation in HeLa cells following a 24 h treatment with either 0.5% DMSO (control) or 100 μM vincristine. Nuclei were stained with the Hoechst solution (grey), G4 structures with BG4 (magenta), and mitochondria with MitoTracker (green). Merged channels are also reported. Scale bar: 5 μm. Quantification showing (**b**) the number of mitochondrial G4 structures and (**c**) the colocalization area of BG4 and MitoTracker signals. (**d**) Colocalization between BG4 and MitoTracker signals was also quantified using Mander’s coefficient. Histograms show the mean ± SD of two independent experiments. An average of 100 cells were screened per condition. The statistical significance was calculated using a Student’s *t*-test on GraphPad Prism 10.2.1 (****: *p* < 0.0001). Refer to Figure S4 for a larger field image and colocalization details.

**Figure 6:**
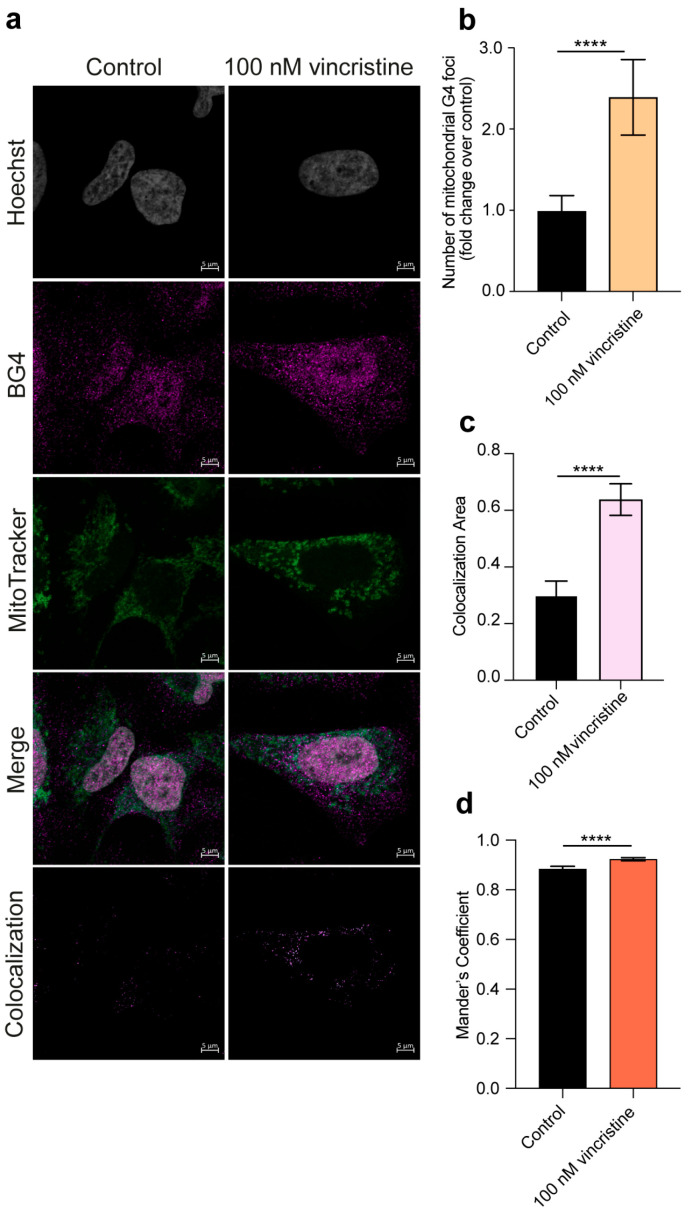
(**a**) Representative immunofluorescence images showing G4 foci formation in U2OS cells following a 24 h treatment with either 0.5% DMSO (control) or 100 nM vincristine. Nuclei were stained with the Hoechst solution (grey), G4 structures with BG4 (magenta), and mitochondria with MitoTracker (green). Merged channels are also reported. Scale bar: 5 μm. Quantification showing (**b**) the number of mitochondrial G4 structures and (**c**) the colocalization area of BG4 and MitoTracker signals. (**d**) Colocalization between BG4 and MitoTracker signals was also quantified using Mander’s coefficient. Histograms show the mean ± SD of two independent experiments. An average of 100 cells were screened per condition. The statistical significance was calculated using a Student’s *t*-test on GraphPad Prism 10.2.1 (****: *p* < 0.0001). Refer to Figure S5 for a larger field image and colocalization details.

**Figure 7:**
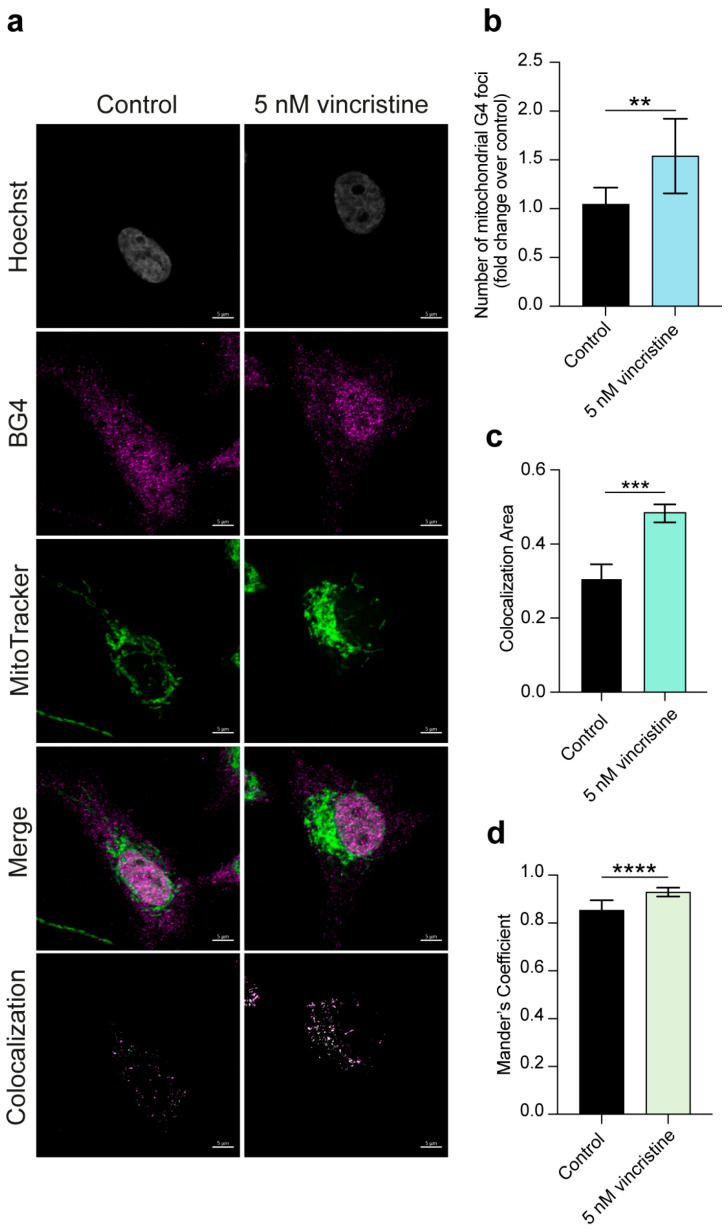
(**a**) Representative immunofluorescence images showing G4 foci formation in MDA-MB-231 cells following a 24 h treatment with either 0.5% DMSO (control) or 5 nM vincristine. Nuclei were stained with the Hoechst solution (grey), G4 structures with BG4 (magenta), and mitochondria with MitoTracker (green). Merged channels are also reported. Scale bar: 5 μm. Quantification showing (**b**) the number of mitochondrial G4 structures and (**c**) the colocalization area of BG4 and MitoTracker signals. (**d**) Colocalization between BG4 and MitoTracker signals was also quantified using Mander’s coefficient. Histograms show the mean ± SD of two independent experiments. An average of 100 cells were screened per condition. The statistical significance was calculated using a Student’s *t*-test on GraphPad Prism 10.2.1 (**: *p* < 0.01; ***: *p* < 0.001; ****: *p* < 0.0001). Refer to Figure S6 for a larger field image and colocalization details.

Importantly, given the significant extranuclear contribution to the observed G4 foci increase and in light of the growing importance of G4s in mitochondrial function and integrity [22,23], we explored whether vincristine could affect the formation of mitochondrial G-quadruplexes. This was achieved through an additional immunofluorescence experiment employing the BG4 antibody and the MitoTracker dye for mitochondria labeling (see Materials and Methods (Section 3) for details) [10,24]. Strikingly, such analysis revealed a remarkable, nearly twofold, increase in the number of mitochondrial G4s in HeLa and U2OS cells (Figure 5 and Figure 6, respectively) and a significant but less pronounced increase in MDA-MB-231 cells (Figure 7), suggesting that vincristine can promote G4 folding within the mitochondrial DNA.

Finally, a portion of the observed signal was also found to be attributable to RNA G4 structures, as pre-treatment with RNase A abolished the vincristine-induced increase in G4 foci, albeit to varying extents (Figures S7 and S8).

Overall, our findings delineate a significant and varied impact of vincristine on the cellular G4 dynamics of three different cancer cell lines, highlighting a previously unappreciated aspect of its anti-cancer activity and potentially expanding our understanding of its mechanism of action, which clearly warrants further investigation.

### 2.2. Analysis of Mitochondrial Structure and Function

Building upon the intriguing effect of vincristine on mitochondrial G4s, we sought to investigate the drug’s impact on mitochondrial morphology and activity, as a potential G4-related consequence. To this end, we performed additional live-cell imaging experiments using MitoTracker DeepRed FM to visualize morphology and Tetramethylrhodamine Ethyl Ester (TMRE) to assess membrane potential. These analyses were carried out exclusively in HeLa and U2OS cells, as a more pronounced increase in mitochondrial G4s upon vincristine treatment was consistently observed in these two cell lines, compared to MDA-MB-231 (see Materials and Methods (Section 3) for details).

**Figure 8:**
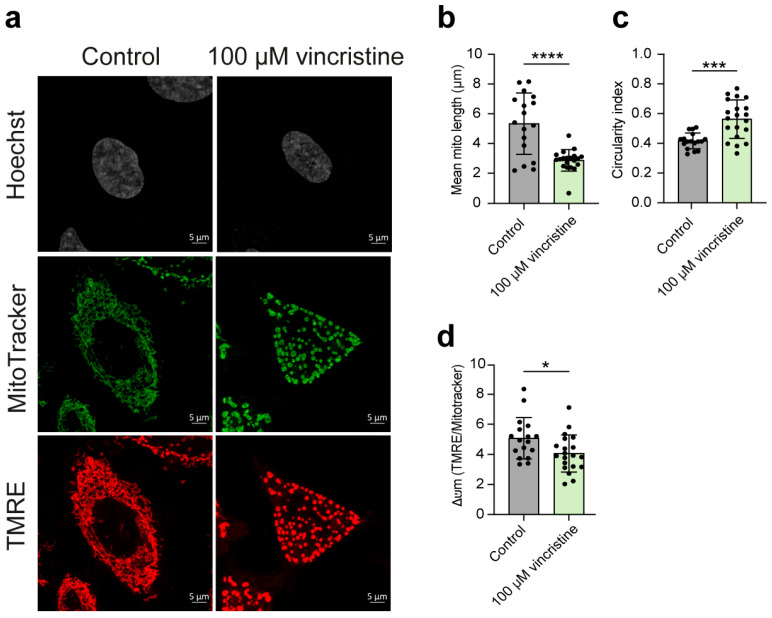
(**a**) Representative images showing HeLa cells treated with either vehicle (0.5% DMSO) or 100 μM vincristine and stained with both MitoTracker DeepRed FM and Tetramethylrhodamine, Ethyl Ester (TMRE) (scale bar: 5 μm). Nuclei were stained with the Hoechst solution (grey) whereas mitochondria with MitoTracker (green) and TMRE (red). (**b**,**c**) Analysis of mitochondrial morphological parameters, including mean mitochondrial length and circularity index. (**d**) Δψm analysis calculated as TMRE/Mitotracker ratio per cell, indicative of mitochondrial membrane polarization and likely mitochondrial functionality. Statistical analysis was computed using a Student’s *t*-test on GraphPad Prism 10.2.1 (*: *p* < 0.05; ***: *p* < 0.001; ****: *p* < 0.0001). Black dots represent mean data for each acquired field.

**Figure 9:**
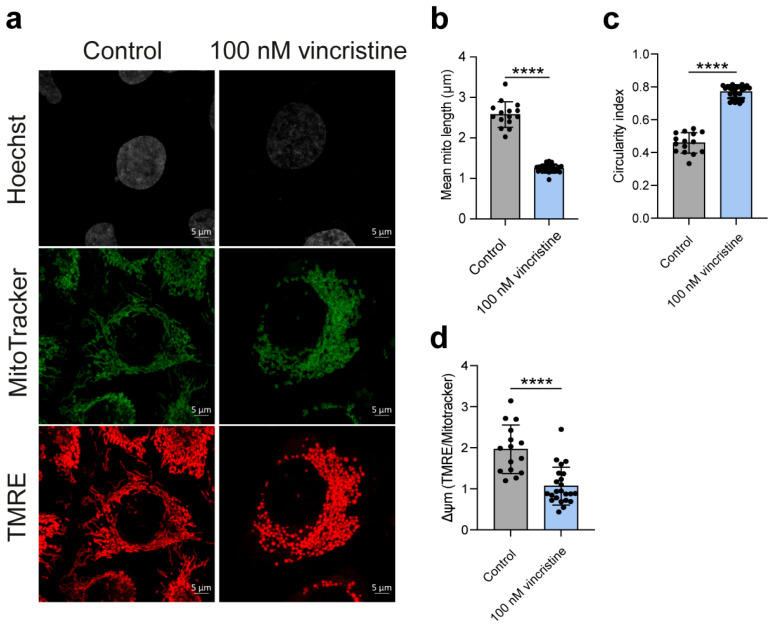
(**a**) Representative images showing U2OS cells treated with either vehicle (0.5% DMSO) or 100 nM vincristine and stained with both MitoTracker DeepRed FM and Tetramethylrhodamine, Ethyl Ester (TMRE) (scale bar: 5 μm). Nuclei were stained with the Hoechst solution (grey) whereas mitochondria with MitoTracker (green) and TMRE (red). (**b**,**c**) Analysis of mitochondrial morphological parameters, including mean mitochondrial length and circularity index. (**d**) Δψm analysis calculated as TMRE/Mitotracker ratio per cell, indicative of mitochondrial membrane polarization and likely mitochondrial functionality. Statistical analysis was computed using a Student’s *t*-test on GraphPad Prism 10.2.1 (****: *p* < 0.0001). Black dots represent mean data for each acquired field.

Strikingly, our analysis revealed that vincristine exposure could lead to a pronounced shift from an elongated mitochondrial network to a more fragmented, rounded morphology in both cell lines. This was quantitatively confirmed by a decrease in the mean mitochondrial length and an increase in the circularity index, a shape descriptor where values closer to 1 indicate more rounded structures (Figure 8b,c and Figure 9b,c) [25,26]. Furthermore, such structural changes were accompanied by a significant decrease in mitochondrial membrane potential, suggesting a marked reduction in mitochondrial functional activity (Figure 8d and Figure 9d) [27].

Overall, our results indicate that vincristine can disrupt the delicate balance of mitochondrial fission-fusion dynamics [22], an effect not previously documented. Such disruption leads to the observed structural and functional decay, potentially contributing to the drug’s established antitumor effect.

### 2.3. Nuclear Magnetic Resonance

To explore whether the results observed in cell-based assays reflected a direct interaction of vincristine with G-quadruplex (G4) DNA structures, we performed a series of biophysical in vitro experiments starting with one-dimensional proton nuclear magnetic resonance (^1^H-NMR) spectroscopy. Specifically, we conducted titration experiments, a widely accepted approach to structurally characterize small molecule–G4 interactions [28,29]. This method monitors chemical shift perturbations (CSPs) in diagnostic proton resonances of nucleotides, particularly imino and aromatic protons, which are sensitive indicators of ligand binding. The pattern and magnitude of CSPs provide insights into the binding site, interaction mode, and conformational preferences of the ligand.

As a preliminary proof-of-concept, we selected three well-characterized G4-forming oligonucleotides: the *c-KIT2* promoter (PDB: 2KYP) [30], the c-*MYC* promoter (PDB: 1XAV) [31], and the human telomeric sequence (mutTel24) (PDB: 2GKU) [32], as structural models representative of the most common G4 folding topologies. Furthermore, a canonical double-stranded DNA (dsDNA) control (see Materials and Methods (Section 3)) was also employed. The dsDNA duplex was included to assess vincristine’s selectivity for G4 versus B-form DNA, an important consideration in anticancer drug development, where off-target interactions with duplex DNA often lead to undesirable cytotoxicity.

The selection of this restricted panel of G4-forming oligonucleotides was guided by: (i) the availability of high-resolution NMR structures; (ii) NMR assignments essential for reliable interpretation of chemical shift perturbations, and (iii) the limited diversity of G4 topologies, with key structural features.

The three chosen G4 sequences are known to adopt distinct topologies: the telomeric sequence forms a hybrid-type G4, whereas c-*KIT2* and c-*MYC* fold into parallel G4 structures [33]. Under the experimental conditions employed, the NMR spectrum of each G4 sample exhibited 12 well-resolved imino proton signals, consistent with the formation of a single, stable G4 conformation comprising three stacked G-tetrads [34,35,36].

Titration experiments were conducted by gradually increasing the molar ratio of vincristine to DNA, up to a final concentration of 8 molar equivalents. This strategy enabled us to investigate whether additional or cooperative binding events might emerge at higher ligand concentrations, as reflected by changes in chemical shift perturbations (CSPs). Notably, vincristine induced distinct, localized CSPs in both c-*KIT2* and c-*MYC* G4-forming sequences (Δδ values in Hz are reported in Tables S1 and S2, respectively).

**Figure 10:**
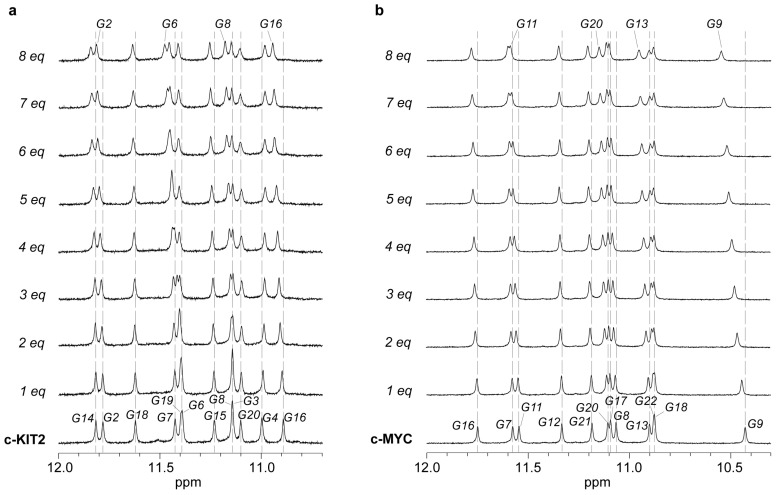
Imino region of the ^1^H-NMR titration spectra for (**a**) c-*KIT2* G4 and (**b**) c-*MYC* G4 upon incremental addition of up to 8 molar equivalents of vincristine at 25 °C. The signals indicated with Gs correspond to the 12 guanine residues involved in the formation of the G-tetrads (numbers refer to the relative PDB structures). Progressive shifts in the imino proton resonances reflect the interaction between vincristine and the G-quadruplex structures.

For the c-*KIT2* G-quadruplex, the most pronounced CSPs, up to ~50 Hz, were observed for the imino proton of G6 (Figure 10a) and the aromatic proton of C1 (Figure S9), both located at the 5′ end of the structure. Additional perturbations at the 5′ end included the imino proton of G2, supporting a consistent pattern of interaction in this region. Smaller CSPs were also detected in the imino protons of G8 and G16 (3′ end), and in the aromatic signals of G4 (3′ end) and T12 (central loop) (Figure S9), indicating possible secondary interactions. These data strongly suggest a preferential binding of vincristine at the 5′ G-quartet.

In the c-*MYC* G-quadruplex, the most significant perturbations, up to ~70 Hz, were observed in the imino proton of G9 (Figure 10b) and the aromatic proton of G22, both located at the 3′ end of the structure (Figure S10, Table S2). Additional CSPs at the 3′ terminus included the imino proton of G13 and the aromatic signal of T23, further supporting a predominant interaction with the 3′ G-quartet and nearby flanking residues. CSPs were also detected at the 5′ end, involving the imino protons of G11 and G20, as well as aromatic signals from G16 and A6, with the latter being a 5′ flanking base.

Taken together, these results indicate that vincristine interacts with both terminal G-quartets in the two G4-forming sequences, although with distinct binding preferences: in c-*KIT2*, vincristine preferentially binds the 5′ G-quartet, whereas in c-*MYC*, the compound shows a slight preference for the 3′ terminal G-quartet and its flanking region. This differential recognition may be due to the structural differences between the two G4 models investigated. Although the observed CSPs were relatively modest, they were reproducible and spatially localized, suggesting, in any case, a specific and reversible interaction at the 5′ and 3′ terminal tetrads.

In contrast, the human telomeric G4 (mutTel24) sequence did not exhibit any detectable chemical shift changes in either the imino or aromatic regions (Figure S11). Likewise, no CSPs were observed for the dsDNA duplex (Figure S12).

Altogether, these findings indicate that vincristine interacts with G4 structures present in the promoter regions of c-*MYC* and c-*KIT2*, but not with the telomeric G4 or the dsDNA duplex. However, given that only three G4 sequences were tested, two parallel and one hybrid, we cannot draw definitive conclusions regarding vincristine’s topological selectivity for G4 structures.

### 2.4. Circular Dichroism Experiments

Following the identification of a direct interaction between vincristine and two of the three G4 sequences investigated (c-*KIT2* and c-*MYC*), we further explored the functional consequences of this binding. In particular, we assessed whether vincristine could influence G4 structural topology and enhance the thermal stability of these DNA structures.

We first performed circular dichroism (CD) spectroscopy to evaluate possible alterations in G4 folding topology upon vincristine binding. CD spectroscopy is a well-established technique for probing G4 conformation, as distinct G4 topologies exhibit characteristic CD signatures [36,37,38]. Parallel G4 structures are typically identified by a positive band near 264 nm and a negative band near 240 nm, reflecting their unique strand orientation and guanine stacking geometry.

To assess the potential effect of vincristine on the G4 topology, CD spectra of DNA–ligand mixtures were recorded after addition of both 4 and 8 molar equivalents of vincristine to the pre-folded G4 sequences. These concentrations were chosen to ensure a ligand excess relative to potential binding sites. However, both for c-*KIT2* and c-*MYC*, the diagnostic CD bands remained essentially unchanged in the presence of both 4 and 8 molar equivalents of vincristine, indicating that the overall G4 topology was preserved upon ligand binding (Figure S13). Minor variations in the 220–240 nm and ~300 nm regions were observed in both cases; however, these signals are attributable to vincristine itself, as confirmed by the CD spectrum of the free compound (Figure S14). These results suggest that vincristine interacts with the G4 structures without inducing significant conformational rearrangements, supporting a binding mechanism that is topology-compatible rather than topology-disruptive.

After confirming that the G4 topology remained unaltered upon vincristine binding, we proceeded to investigate whether the ligand could enhance the thermal stability of the G4 structures through CD melting experiments. This technique allows the monitoring of G4 unfolding as a function of temperature, with the melting temperature (*T_m_*) representing the midpoint of the transition from folded to unfolded state. Melting profiles were recorded in the absence of vincristine to establish baseline *T_m_* values. Under these conditions, the c-*KIT2* G4 displayed a melting temperature of 59 °C (±1 °C), while the c-*MYC* G4 showed a *T_m_* of 78 °C (± 1 °C). Subsequently, the same experiments were performed in the presence of 4 and 8 molar equivalents of vincristine to assess any ligand-induced thermal stabilization.

As shown in Supplementary Materials (Figure S15), the addition of vincristine resulted in only marginal increases in the *T_m_* values, in the range of 1–2 °C, for both G4 sequences. However, these variations fall within the experimental error margin commonly associated with this technique and therefore cannot be considered statistically significant.

### 2.5. Isothermal Titration Calorimetry

To further characterize the interaction between vincristine and G4 structures at the thermodynamic level, we performed isothermal titration calorimetry (ITC) experiments using the c-*KIT2* and c-*MYC* G4-forming sequences. ITC is a powerful and widely employed technique to dissect the energetics of ligand–G-quadruplex recognition, providing direct measurements of heat released or absorbed upon binding, and thereby enabling the determination of enthalpic contributions (ΔH°) to complex formation.

**Figure 11:**
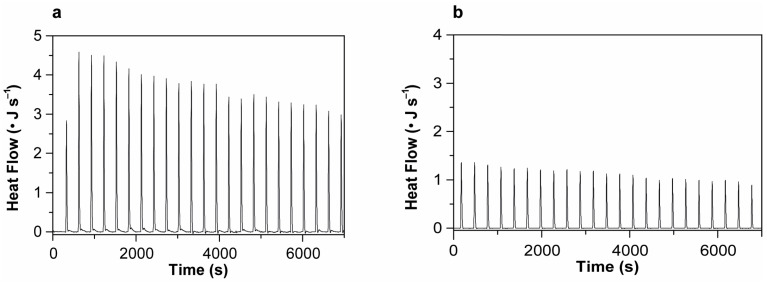
Calorimetric peaks from the isothermal titration of (**a**) c-*KIT2* and (**b**) c-*MYC* G-quadruplex DNA with vincristine (8 molar equivalents) at 25 °C. The heat released upon each injection reflects the enthalpy of interaction between vincristine and the folded G4 structures.

The titration experiments were carried out at 25 °C, by incrementally injecting vincristine (up to 8 molar equivalents) into a calorimetric cell containing folded G4 DNA. Each injection generates a heat signal corresponding to the interaction between the ligand and DNA, which is integrated over time to yield a calorimetric peak. The area under each peak was used to compute the ΔH° values. The peaks obtained were homogeneous in shape and magnitude prior to saturation, allowing reliable integration and averaging (Figure 11). To exclude non-specific contributions, a control titration of vincristine into DNA-free buffer was also conducted under identical conditions (Figure S16), confirming that the observed thermal signals were solely attributable to specific interactions with DNA.

Although the complexity of the binding process, likely involving multiple weak and/or heterogeneous interaction sites, prevented a robust determination of the binding constant (K_b_) and stoichiometry (n), the ITC data provided clear and reproducible enthalpy values for each DNA-ligand pair. In both cases, the measured ΔH° values were negative, consistent with exothermic and enthalpically driven interactions. Negative enthalpies typically reflect the formation of favorable non-covalent interactions, such as hydrogen bonding, van der Waals contacts, and π–π stacking, which are known to underlie many G4–ligand binding events.

Notably, the interaction enthalpy for *c-KIT2* G4 was substantially more negative than that for c-*MYC* G4 (Table 1), suggesting that vincristine forms more numerous and/or stronger stabilizing contacts with c-*KIT2* G4. This observation is in line with previous studies showing that structural features such as loop orientation and groove accessibility can significantly influence the thermodynamics of G4–ligand interactions [39].

These findings support a scenario in which vincristine engages in weak but energetically favorable binding to G4 structures, with a higher affinity for the c-*KIT2* G4.

### 2.6. Molecular Modeling

After employing a range of biophysical techniques to investigate the interaction between vincristine and the G-quadruplex structures formed in the promoter regions of c-*KIT2* and c-*MYC*, we sought to complement these findings with a structural model of the binding modes. The aim was to generate a visual and mechanistic representation of vincristine’s interaction with the G4 scaffolds, offering a molecular-level rationale for the chemical shift perturbations and binding preferences observed experimentally. To this end, molecular docking simulations were performed using available NMR-resolved G-quadruplex structures, providing detailed insights into the spatial and energetic features of the interaction.

#### 2.6.1. c-*KIT2* Gene Promoter

**Figure 12:**
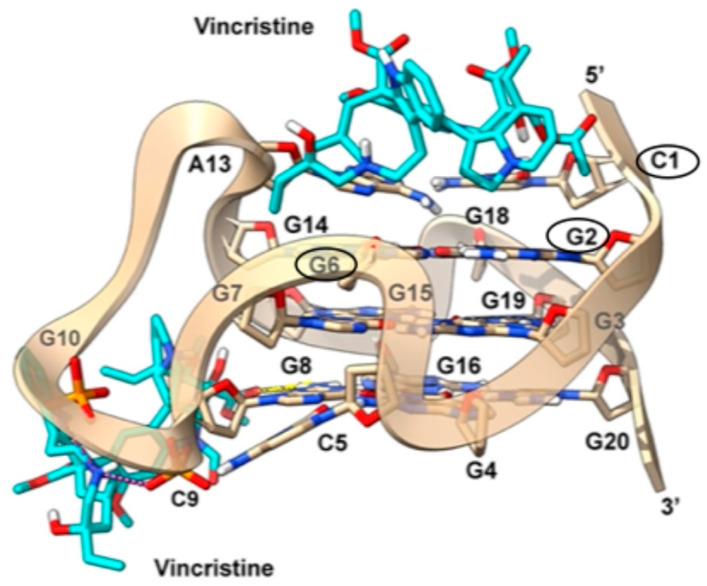
Docking poses of vincristine at the 5′ and 3′ ends of the c-*KIT2* gene promoter G4 structure (PDB code: 2KYP). The ligand is shown as cyan sticks, while the c-*KIT2* G4 is depicted as a light brown transparent cartoon and sticks. Non-polar hydrogens are omitted for clarity. Hydrogen bonds and salt bridges are shown, respectively, as yellow and magenta dashed lines. Bases circled in black correspond to those exhibiting the strongest chemical shift perturbations in the NMR titration with vincristine.

To investigate the interaction between vincristine and the parallel G-quadruplex structure of the c-*KIT2* promoter (PDB code: 2KYP), we employed an Induced Fit Docking (IFD) procedure. This approach allowed for flexibility in C1 and A3 at the 5′ end and T21 at the 3′ end, which might otherwise hinder the binding of a bulky ligand such as vincristine (see Materials and Methods (Section 3) for details). At the 5′ end, the best docking pose shows vincristine positioned above the terminal G-tetrad, forming cation-π interactions with G2 and G6. Specifically, the vindoline unit of vincristine engages in a cation-π interaction with G2 via the tetrahydropyridine nitrogen, while the catharanthine moiety establishes a similar interaction with G6 through its piperidine nitrogen. Additionally, vincristine forms a hydrogen bond between its vindoline hydroxyl group and the carbonyl oxygen of C1, further stabilizing its binding at the 5′ end. These contacts closely align with the most pronounced NMR CSPs detected experimentally at G6 and C1, which are circled in black in Figure 12, confirming that the 5′ terminus represents the primary binding site.

At the 3′ end, docking identified a single pose in which vincristine partially aligns with experimental data, inserting into the groove created by the G8-G14 loop. Here, the ligand establishes a hydrogen bond with the exocyclic amino group of G8. Further stabilization is provided by salt bridges: one between the piperidine nitrogen of the catharanthine moiety and the phosphate groups of C9 and G10, and another between the tetrahydropyridine nitrogen of the vindoline moiety and the phosphate of G15, which is also engaged in a hydrogen bond with the vindoline hydroxyl group. Importantly, these binding modes are in good agreement with smaller perturbations observed for this region in the NMR titrations, particularly in terms of vincristine’s primary stabilization at the 5′ end and its weaker interaction at the 3′ end, supporting the proposed mechanism of G-quadruplex binding.

#### 2.6.2. c-*MYC* Gene Promoter

**Figure 13:**
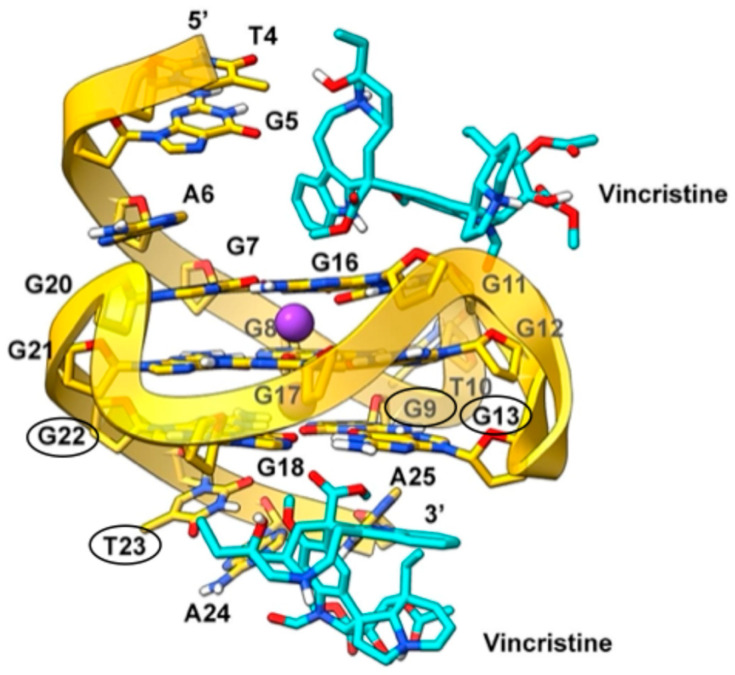
Docking poses of vincristine at the 5′ and 3′ ends of the c-*MYC* gene promoter G4 structure (PDB code: 5W77). The ligand is shown as cyan sticks, while the c-*MYC* G4 is depicted as a yellow transparent cartoon and sticks. Non-polar hydrogens are omitted for clarity. Bases circled in black correspond to those exhibiting the strongest chemical shift perturbations in the NMR titration with vincristine.

To investigate the binding mode of vincristine to the c-*MYC* gene promoter, we performed docking calculations using the G4 structure from the PDB entry 5W77. In particular, blind docking encompassing the entire G4 structure was carried out, based on the binding promiscuity of vincristine revealed by NMR experiments. In agreement with these data, blind docking results revealed multiple binding poses along the G-quadruplex. Indeed, based on NMR experimental data identifying preferred binding regions, we selected the poses that best matched them. At the 5′ end, vincristine is positioned between the lower tetrad and the flanking triplet (T4, G5, A6). In this region, the ligand appears to form π-π stacking interactions with G11, circled in black in Figure 13. At the 3′ end, corresponding to the region that displayed the strongest chemical shift perturbations in the NMR titration (see Table S2 and Figure 10), the most favorable poses show vincristine positioned between the upper tetrad and the triplet of flanking bases (T23, A24, A25). Here, the indole moiety of the catharanthine unit is oriented parallel to G13 of the lower tetrad, allowing for the formation of a π-π interaction with this residue (Figure 13).

Overall, the molecular modeling results are consistent with the experimental data and support the hypothesis that vincristine engages the terminal G-quartets of G-quadruplex structures. In c-*KIT2*, the binding appears more localized and stable at the 5′ end, while in c-*MYC*, a more distributed interaction pattern is observed, with a slight preference for the 3′ end and its flanking residues. These structural models reinforce the conclusions drawn from NMR titration experiments and provide a framework for interpreting the modest biophysical effects observed in vitro.

## 3. Materials and Methods

### 3.1. Materials

Dulbecco’s modified Eagle’s medium (DMEM), Dulbecco’s phosphate-buffered saline (PBS), fetal bovine serum (FBS), penicillin and streptomycin for cell culture were obtained from Euroclone S.p.A (Pero, Italy). Vincristine was supplied by Merck (St Louis, MO, USA), along with the Hoechst 33,258 solution and Mowiol 4-88.

Dimethyl sulfoxide (DMSO), 4% paraformaldehyde, Triton X-100, and bovine serum albumin (BSA) were purchased from HiMedia (Kennett Square, Pennsylvania, USA).

The Ambion^TM^ RNase A solution (1 mg/mL), the MitoTracker dyes for mitochondria labeling, TMRE and NucBlue™ Live ReadyProbes™ Reagent for live-cell experiments were obtained from Thermo Fisher Scientific (Waltham, MA, USA). The µ-Slide 18-well plates were supplied by ibidi GmbH (Gräfelfing, Germany).

### 3.2. Cell Culture

Human cervical adenocarcinoma (HeLa), human breast cancer cells (MDA-MB-231) and human osteosarcoma (U2OS) cells were grown in Dulbecco’s Modified Eagle Medium (DMEM) supplemented with 10% Fetal Bovine Serum (FBS) and 1% Penicillin/Streptomycin. All cell lines were sub-cultured at 90% confluence in a split ratio of 1:5 every two days and maintained at 37 °C in a humidified atmosphere containing 5% CO_2_.

Vincristine was dissolved in 100% sterile DMSO to make a stock solution at 20 mM concentration and then diluted in cell culture medium to the required concentration before use.

### 3.3. Immunofluorescence Assays

#### 3.3.1. BG4 Immunofluorescence

HeLa, MDA-MB-231 and U2OS cells were seeded on sterile coverslips in a 24-well plate, at a density of 90,000 (HeLa and MDA-MB-231) and 120,000 (U2OS) cells per well, and incubated overnight. Upon attachment, cells received either vincristine (100 μM for HeLa cells, 5 nM for MDA-MB-231 cells and 100 nM for U2OS cells) or vehicle (0.5% DMSO) for 24 h. Afterwards, cells were fixed in 4% paraformaldehyde for 10 min at RT, and then permeabilized with 0.1% Triton X-100 in PBS for an additional 10 min at RT. To block non-specific binding sites, incubation with 5% BSA in PBS for 30 min at RT was performed. Subsequently, cells were stained at RT for 1 h, with the BG4 antibody (#MABE917, Merck-Millipore, Darmstadt, Germany), diluted 1:100 in blocking buffer. Following three washes with PBS, cells were further incubated at RT for 1 h, with the rabbit anti-FLAG antibody (#F7425, Merck), diluted 1:2500 in blocking buffer. After three more rinsing steps with PBS, incubation with the donkey anti-rabbit Alexa Fluor 488-Conjugate secondary antibody (#A-21206; ThermoFisher, Waltham, MA, USA), diluted 1:500 in blocking buffer, for 1 h, at RT was done. Nuclear staining was obtained by using the Hoechst 33258 solution (1:3000, 10 min). Finally, coverslips were rinsed once with distilled water and mounted on microscope slides with Mowiol 4-88.

To investigate the contribution from RNA G-quadruplexes, cells were treated with RNAse A (1 h, at 37 °C) prior to BG4 staining, as reported elsewhere [40].

The data reported in the figures represent the average of at least seven acquired fields, each containing 10–15 cells, across two independent experimental batches.

#### 3.3.2. MitoTracker Staining

HeLa, MDA-MB-231 and U2OS cells were exposed to vincristine or vehicle for 24 h. At the end of the treatments, the growth medium was discarded and cells were incubated with 100 nM MitoTracker dye (#M7512, ThermoFisher) in warm FBS-free medium at 37 °C for 30 min. Next, cells were washed with warm FBS-free medium, fixed in 4% paraformaldehyde for 10 min at room temperature, and then processed for BG4 immunofluorescence as described above.

Image acquisition involved recording z-stacks of three planes using a confocal microscope (Zeiss LSM 980, Zeiss, Oberkochen, Germany, Plan-Apochromat 63×/1.4NA oil objective). For image analysis, a maximum intensity projection was generated from the z-stacks. G4 foci were then segmented and quantified using the Image Analysis package of ZEISS ZEN Blue 3.1 software. Similarly, for MitoTracker staining, both G4 foci and mitochondria were segmented, and their quantities and colocalization were quantified using the same software.

The data reported in the figures represent the average of at least seven acquired fields, each containing 10–15 cells, across two independent experimental batches.

### 3.4. Analysis of Mitochondrial Structure and Function

HeLa and U2OS cells were seeded at a density of 18,000 cells per well in Ibidi µ-Slide 18-well plates and incubated at 37 °C. After 24 h, cells were treated with either a vehicle or vincristine (100 µM for HeLa cells and 100 nM for U2OS cells) for an additional 24 h. For live-cell imaging, cells were stained for 30 min, at 37 °C, with 5 ng of MitoTracker DeepRed FM (#M22426, ThermoFisher), 1 nM TMRE (#T669, ThermoFisher) and NucBlue™ Live ReadyProbes™ Reagent (#R37605, ThermoFisher). The chambers were then located in a dedicated holder for observation with a Zeiss LSM 980 confocal microscope equipped with a Plan-Apochromat 63×/1.4 NA oil objective. Environmental conditions (5% CO_2_ and 95% relative humidity) were maintained throughout the experiment using an ibidi Heating Unit XL S2 with a camera system. All images were processed and analyzed with ZEISS ZEN Blue 3.1 software.

### 3.5. Oligonucleotide Preparation

The oligonucleotide sequences d[TGA(GGGTGGGTA)_2_A] (c-*MYC*, PDB code: 1XAV), d(CGGGCGGGCGCTAGGGAGGGT) (c-*KIT2*, PDB code: 2KYP), d[(TT(GGGTTA)_3_GGGA)] (mutTel24, PDB code: 2GKU), and the complementary duplex-forming pair d(GTA ACT AGT TAA CGA TC) (antisense) and d(GAT CGT TAA CTA GTT AC) (sense) were purchased from Eurogentec (Liège, Belgium) and supplied as dry powders. Oligonucleotides were dissolved in a buffer containing 10 mM KH_2_PO_4_ and 20 mM KCl (pH 7.0), heated to 90 °C for 5 min, and then slowly cooled to room temperature (RT) overnight to allow proper folding. Prior to use in experiments, samples were stored at 4 °C for 24 h.

### 3.6. Nuclear Magnetic Resonance Experiments

One-dimensional proton nuclear magnetic resonance (1D ^1^H-NMR) spectra were acquired at 25 °C, using a Bruker Avance NEO spectrometer (Bruker Biospin GmbH, Rheinstetten, Germany) operating at 600 MHz and equipped with a QCI cryoprobe for 5 mm sample tubes. DNA samples were prepared at a concentration of 0.2 mM, in 0.22 mL of buffer solution (H_2_O/D_2_O 9:1). Vincristine was dissolved in DMSO-d_6_ to prepare a 50 mM stock solution. For each equivalent of vincristine, 1 µL of stock solution was added directly to 250 µL of DNA solution inside the NMR tube. For two sequences (*c-KIT2* and *c-MYC*), titrations were performed up to 8 equivalents, corresponding to a maximum addition of 8 µL of stock (≈3.2% *v*/*v* DMSO in the NMR sample). This concentration is well below the 5% *v*/*v* threshold commonly reported in the literature as non-perturbative for G-quadruplex NMR studies, where such small amounts of DMSO do not cause significant shifts in the imino proton region [41,42]. For the human telomeric G-quadruplex (mutTel24) and a double-helical dsDNA control, titrations were limited to 4 equivalents because no changes in the imino or aromatic signals were observed until this point, indicating no detectable interaction between vincristine and these sequences. NMR spectra were recorded employing the ‘*zgesgp*’ pulse program for water suppression and the following parameters: 64 scans, spectral width 26,0359 Hz, delay 3 s, receiver gain 101, and 32 k points. NMR data was processed using the Topspin 4.4.0 software (Bruker Biospin GmbH, Rheinstetten, Germany).

### 3.7. Circular Dichroism Spectroscopy

Circular Dichroism (CD) spectra were recorded on a Jasco J-815 spectropolarimeter (JASCO Inc., Tokyo, Japan) equipped with a PTC-423S/15 Peltier temperature controller. Samples were prepared in a 10 mM KH_2_PO_4_/K_2_HPO_4_ 20 mM KCl buffer (pH 7.00) at a DNA concentration of 20 µM. The vincristine solution was prepared by dissolving the powder in 100% DMSO to obtain a 10 mM solution. Oligonucleotide/compound mixtures were obtained by adding 4 or 8 molar equivalents of the compound to the folded DNA structures. All the spectra were recorded at 20 and 100 °C in the 230–340 nm wavelength range and averaged over three scans. The scanning speed was 100 nm min^−1^, with a 2 s response and 2 nm bandwidth.

CD melting experiments were carried out in the 20–100 °C temperature range with 1 °C min^−1^ heating rate, following changes in CD signal at the wavelength of the maximum CD intensity (264 nm for both c-*KIT2* and c-*MYC*). The melting curves were normalized between 0 and 1, and the melting temperatures (*T_m_*) were determined using the curve fitting function in OriginPro 2021 software (OriginLab Corp., Northampton, MA, USA).

### 3.8. Isothermal Titration Calorimetry

Isothermal Titration Calorimetry (ITC) measurements were performed using a nano-ITC (TA instruments, New Castle, DE, USA) at 25 °C. Vincristine solution, with concentrations ranging from 300 to 600 μM, was injected into the calorimetric vessel containing a DNA solution (~20 μM), in aliquots of 2 μL with 300 s interval. Dilution heat was evaluated in control experiments in which the vincristine solution was injected into the buffer. For all experiments, the heat flow values were plotted versus time after correction for dilution heat. All measurements were performed in duplicate.

### 3.9. Molecular Modeling

#### 3.9.1. DNA Structure Selection and Preparation

Among the various parallel G4 structures from the c-*MYC* gene promoter available in the Protein Data Bank, we selected the NMR solution structure in complex with the small molecule DC-34 (PDB code: 5W77) [43]. This structure exhibits a 2:1 ligand/DNA stoichiometry, with one ligand molecule interacting with the G-tetrads at either the 5′ or the 3′ ends. Compared to other available structures, 5W77 adopts a more open conformation, where the tetrads are less shielded by loops or stabilizing interactions, providing greater accessibility for vincristine binding. The 5W77 structure contains multiple conformers among which we selected the first one, which was prepared using the Protein Preparation Wizard [44] in Maestro [45]. In particular, hydrogen atoms were added, and protonation states and hydrogen bonding networks were assigned using the PropKa [46] program at physiological pH. Hydrogen positions were finally minimized using the OPLS4 force field [47].

For c-*KIT2*, we selected the only available NMR structure of the G-quadruplex from this gene promoter (PDB code: 2KYP) [30]. Like the c-*MYC* G4 structure, this motif also contains multiple conformers, and our focus was on the first conformer, which was prepared using Maestro’s Protein Preparation Wizard as described above.

#### 3.9.2. Ligand Preparation

Vincristine was designed using the 2D Sketcher tool in Maestro. To explore the conformational space of the ligand, a macrocycle conformational sampling [48] was performed using MacroModel, as part of the Schrodinger Suite [49]. The simulation included 5000 iterations at 1000 K, with each iteration comprising 0.50 ps of high-temperature molecular dynamics followed by annealing. A Large-Scale Low Mode Optimization Dynamics (LLMOD) [50] approach was used, allowing extensive conformational sampling through 5000 steps per search and 30 low-frequency motion modes. Conformational moves ranged from 3 Å to 18 Å, with enhanced planar torsion sampling applied to capture the flexibility of vincristine’s macrocycles. The OPLS4 force field [47] and implicit water solvation were used to simulate physiological conditions. Conformations with an RMSD ≤ 3 Å from the global minimum and within an energy window of 5.0 kcal/mol were selected, resulting in 7 possible conformers. These conformers were prepared using the LigPrep [51] module in Maestro, applying the OPLS4 force field. The tautomeric and protonation states were assigned within the pH 7.4 ± 2.0 range, while the chirality of each conformation was retained from the 3D structures. At the end of the LigPrep process, four different protomeric states were generated for each of the seven conformations of vincristine. To streamline our docking studies, we selected only the most probable protomeric state for each conformation. The most likely state was identified based on the ionization penalty property in Maestro, which evaluates the energetic favorability of different protonation states. According to this analysis, the most probable protomeric state for vincristine was the doubly protonated form.

#### 3.9.3. Molecular Docking Simulations

For the c-*MYC* G4 structure, blind docking was performed using AutoDock4 [52] on the solution NMR structure with PDB code 5W77. A docking box was set to encompass the entire G-quadruplex, ensuring a comprehensive search of potential binding sites. This approach was chosen based on experimental data indicating that vincristine interacts with multiple regions of the quadruplex, with a preference for the tetrads at the 5′ and 3′ ends. The atomic affinity maps were generated using AutoGrid4 with the standard AutoDock4 forcefield. The center of the search space was set to x = 3.1, y = 0.572, and z = −4.458, using PDB 5W77 as a reference, and grid box was sized to 90 × 80 × 76 points. The grid spacing was set to 0.375 Å, and smoothing was applied at 0.5 Å. All dockings were performed using a Lamarckian Genetic Algorithm (LGA) [53], with 10 individual LGA runs (each LGA run produces one pose). A population size of 150 was used, with a maximum of 10,000,000 evaluations and up to 27,000 generations. Other search parameters were used with their default values.

As concerns the G4 of the c-*KIT2* gene promoter, we performed Induced Fit Docking (IFD) calculations [54,55] with Maestro, using the NMR solution structure with PDB code 2KYP. Experimental data suggest that vincristine interacts primarily with the 5′ and 3′ G-tetrads, as well as with the loop connecting G14 and G8. However, ligand access to these regions is hindered by C1 and A3 at the 5′ end and T21 at the 3′ end. Thus, we performed two different sets of calculations, centering the docking box either on the 5′ (G2-G6-G14-G18) and 3′ (G4-G8-G16-G20) G-tetrads. During the rigid docking phase (Glide Docking), the side chains of C1 and A3 at the 5′ end and T21 at the 3′ end were trimmed to facilitate ligand binding. In the refinement phase (Prime Refinement), we refined the residues within 5.00 Å of the docking poses, manually adding C1, A3, and T21 to the refinement list. To preserve the structural integrity of the G4, all guanines forming the tetrads were constrained to prevent any distortion. The poses that best aligned with experimental data were selected for further consideration.

## 4. Conclusions

This study provides proof-of-concept evidence that vincristine perturbs the G-quadruplex (G4) landscape and mitochondrial physiology in cancer cells, adding a new layer to its established role as a microtubule-targeting drug. Using HeLa, MDA-MB-231, and U2OS cell lines, we observed a reproducible increase in cellular G4 foci after vincristine treatment, with mitochondrial G4s elevated by ~1.5–2.5 fold compared to untreated controls. This was accompanied by a significant reduction in mitochondrial membrane potential, suggesting that G4 modulation may contribute to organelle dysfunction and cell death. Importantly, these findings provide a mechanistic context for our previous work showing a pronounced synergistic cytotoxic effect when vincristine was combined with the canonical G4 stabilizer RHPS4. The present data support the idea that vincristine can influence G4 biology, and that additional stabilization by a dedicated ligand exacerbates this effect, thereby strengthening the hypothesis that G4 engagement contributes to vincristine’s cytotoxic profile. Biophysical assays on representative G4-forming sequences further indicated that vincristine interacts with G4 DNA through weak and energetically favorable contacts, without altering overall topology.

Future work, including genome-wide transcriptomic analyses, functional modulation of G4 dynamics, and in vivo studies, will be required to dissect the downstream molecular consequences of vincristine-induced G4 modulation, thus clarifying the quantitative contribution of G4 perturbation to vincristine cytotoxicity.

## Figures and Tables

**Table 1:** Enthalpy change values obtained from DNA titrations with vincristine.

+ Vincristine	Δ*H*° (kJmol^−1^) *
c-*KIT2*	−52.8 ± 5.8
c-*MYC*	−11.7 ± 2.0

* The error on ΔH° is ±10%.

## Data Availability

The original contributions presented in this study are included in the article/Supplementary Materials. Further inquiries can be directed to the corresponding authors.

## Supplementary Materials

The following supporting information can be downloaded at https://www.mdpi.com/article/10.3390/ijms26199606/s1.

## Abbreviations

The following abbreviations are used in this manuscript:

BG4	G-quadruplex-specific antibody
BSA	Bovine serum albumin
CD	Circular dichroism
c-*KIT2*	Gene encoding the receptor tyrosine kinase protein
c-*MYC*	Cellular *MYC* proto-oncogene
DMEM	Dulbecco’s modified Eagle medium
DMSO	Dimethyl sulfoxide
dsDNA	Double-stranded DNA
FBS	Fetal bovine serum
G4	G-quadruplex
HeLa	Human cervical cancer cell Line
ITC	Isothermal titration calorimetry
mtDNA	Mitochondrial DNA
NMR	Nuclear magnetic resonance
PBS	Phosphate-buffered saline
PDB	Protein data bank
rG4	RNA G-quadruplex
RNase A	Ribonuclease A
TMRE	Tetramethylrhodamine Ethyl Ester
UTR	Untranslated region
U2OS	Human osteosarcoma cell line
