# Supplementary material for: Vincristine Beyond Mitosis: Uncovering a First Link to G-Quadruplex DNA in Cancer Cells
Source: Int J Mol Sci. 2025 Oct 1;26(19):9606. doi: 10.3390/ijms26199606 (PMC12524732; doi:10.3390/ijms26199606)
Supplement: Supplementary file 1 [file ijms-26-09606-s001.zip › ijms-3810338-supplementary.pdf]

Supplementary Material

Vincristine Beyond Mitosis: Uncovering a First Link to G-Quadruplex DNA in Cancer Cells

Anna Di Porzio [1,†], Carolina Persico [1,†], Francesca Romano [1], Alessandra Barra [1], Immacolata Aiello [1], Ludovica D'Auria [2], Sara Abate [1], Federica D'Aria [1], Concetta Giancola [1], Elpidio Cinquegrana [1], Francesco Saverio Di Leva [1], Jussara Amato [1], Simona Marzano [1], Nunzia Iaccarino [1,*] and Antonio Randazzo [1,*]

[1] Department of Pharmacy, University of Naples Federico II, Via D. Montesano 49, 80131 Naples, Italy; anna.diporzio@unina.it (A.D.P.); carolina.persico@unina.it (C.P.); francesca.romano2@unina.it (F.R.); aless.barra@studenti.unina.it (A.B.); imma.aiello@studenti.unina.it (I.A.); sar.abate@studenti.unina.it (S.A.); federica.daria@unina.it (F.D.); giancola@unina.it (C.G.); elpidio.cinquegrana@unina.it (E.C.); francesco.dileva@unina.it (F.S.D.L.); jussara.amato@unina.it (J.A.); simona.marzano@unina.it (S.M.)

[2] CEINGE—Biotecnologie Avanzate Franco Salvatore, 80145 Naples, Italy; daurial@ceinge.unina.it

* Correspondence: nunzia.iaccarino@unina.it (N.I.); antonio.randazzo@unina.it (A.R.)

† These authors contributed equally to this work.

Table of contents **Page**

Figure S1……S2
Figure S2……S3
Figure S3……S4
Figure S4……S5
Figure S5……S6
Figure S6……S7
Figure S7……S8
Figure S8……S9
Figure S9……S10
Figure S10……S11
Figure S11……S12
Figure S12……S13
Figure S13……S14
Figure S14……S15
Figure S15……S16
Figure S16……S17
Table S1……S18
Table S2……S19

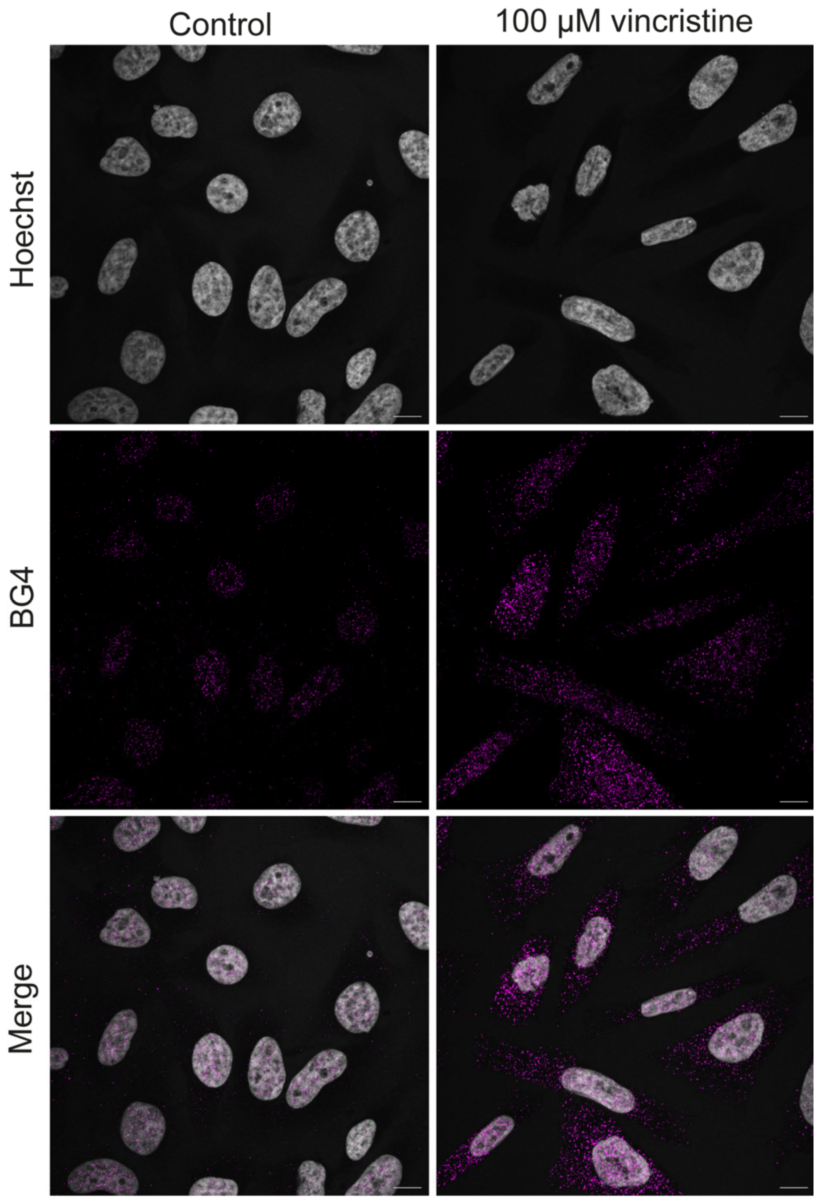

Figure S1. Representative immunofluorescence images showing G4 foci formation in HeLa cells treated for 24 h with either 0.5 % DMSO (control) or 100 µM vincristine. Nuclei were stained with the Hoechst solution (grey) and G4 structures with BG4 (magenta). Merged channels are also reported. Scale bar: 5 µm.

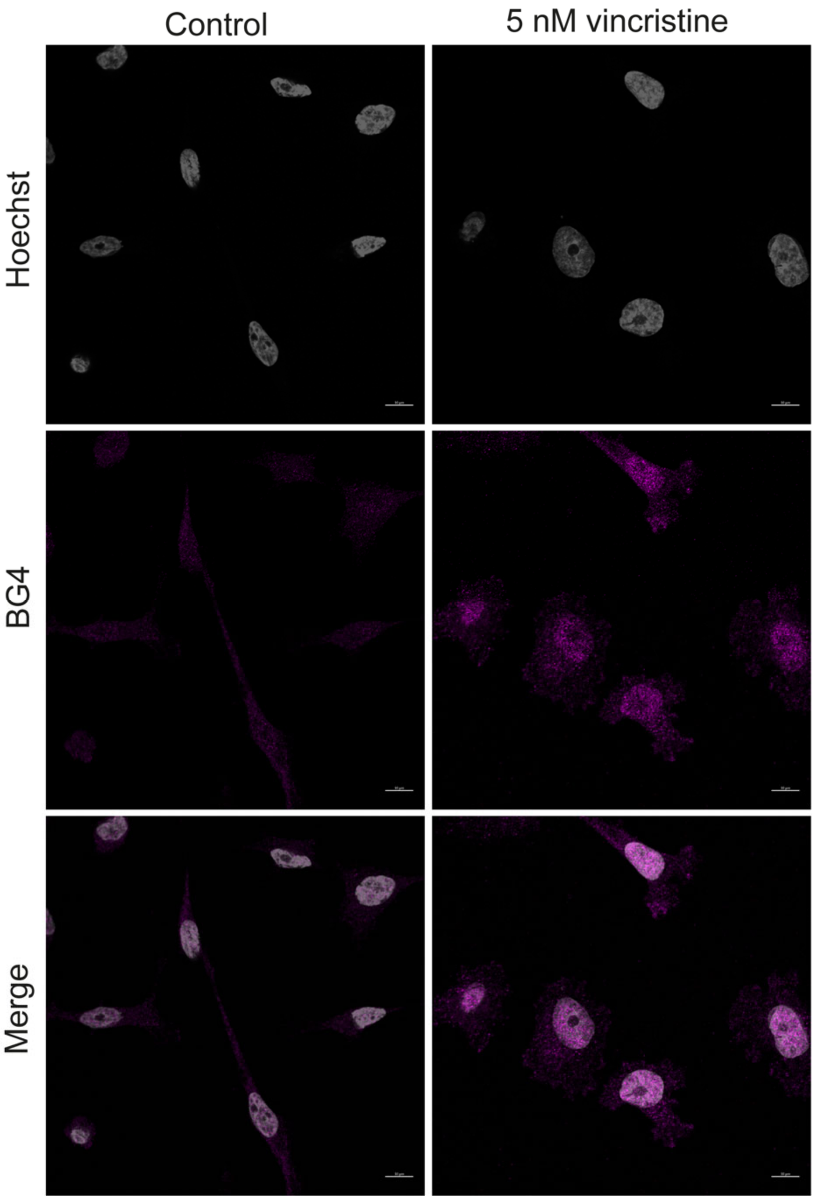

Figure S2. Representative immunofluorescence images showing G4 foci formation in MDA-MB-231 cells treated for 24 h with either 0.5 % DMSO (control) or 5 nM vincristine. Nuclei were stained with the Hoechst solution (grey) and G4 structures with BG4 (magenta). Merged channels are also reported. Scale bar: 5 μm.

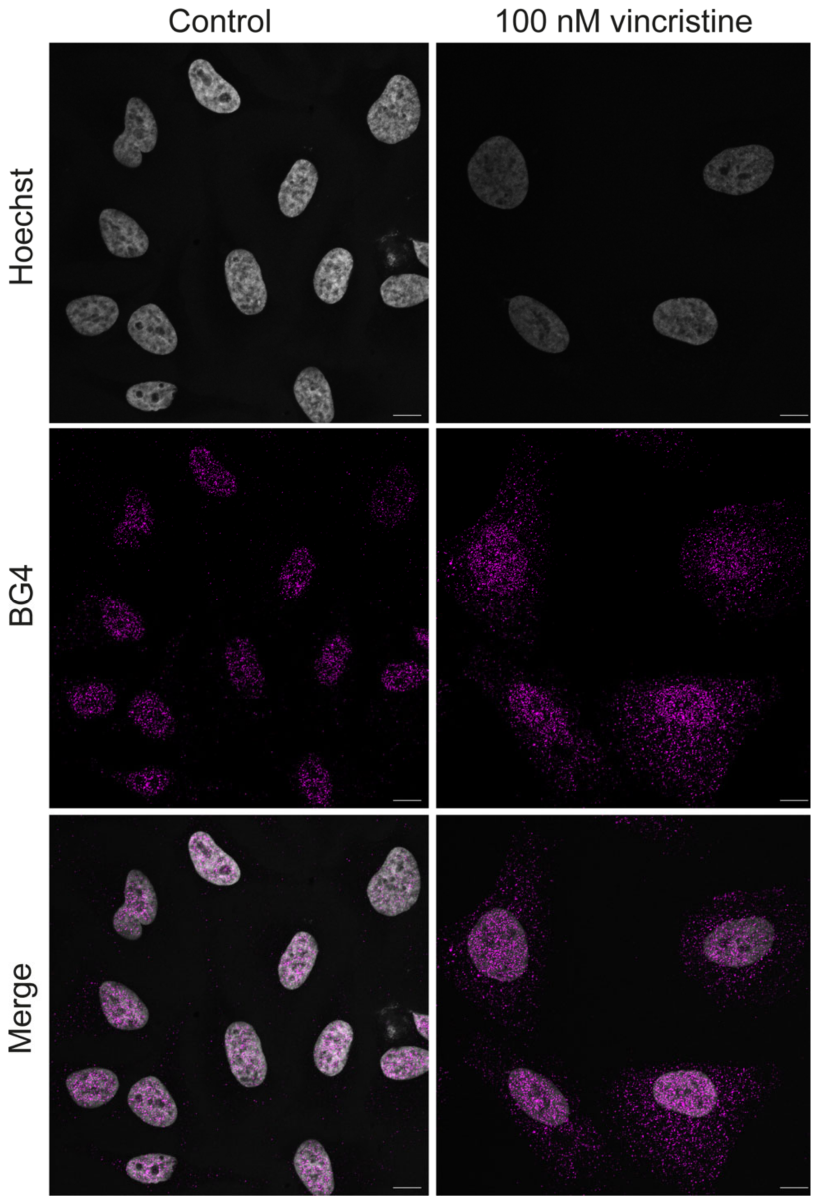

Figure S3. Representative immunofluorescence images showing G4 foci formation in U2OS cells treated for 24 h with either 0.5 % DMSO (control) or 100 nM vincristine. Nuclei were stained with the Hoechst solution (grey) and G4 structures with BG4 (magenta). Merged channels are also reported. Scale bar: 5 μm.

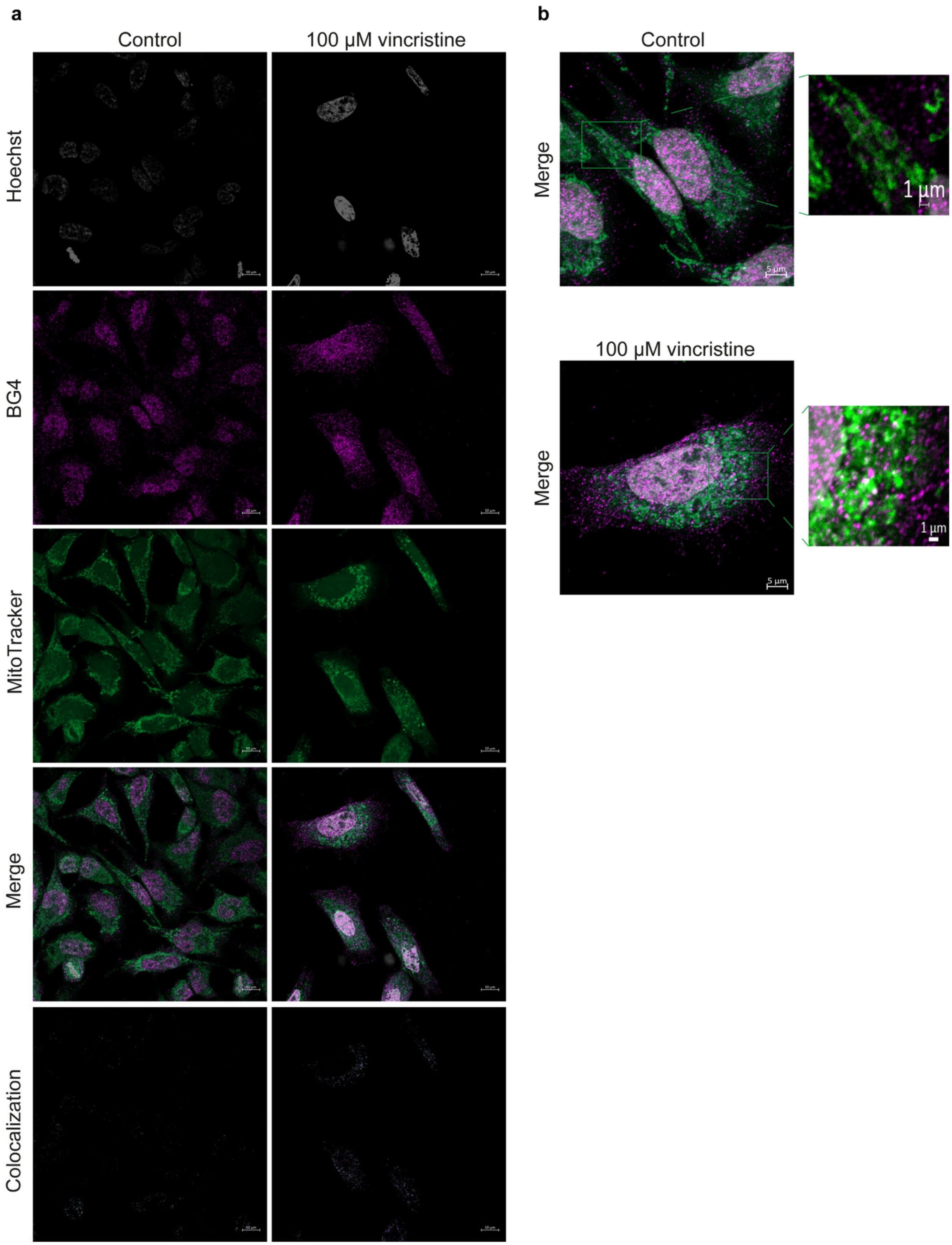

Figure S4. (**a**) Representative immunofluorescence images showing G4 foci formation in HeLa cells following a 24-hour treatment with either 0.5 % DMSO (control) or 100 μM vincristine. Nuclei were stained with the Hoechst solution (grey), G4 structures with BG4 (magenta), and mitochondria with MitoTracker (green). Merged channels and colocalization areas are also reported. Scale bar: 10 μm. (**b**) Colocalization details (scale bar: 5 μm for lower magnification, 1 μm for higher magnification).

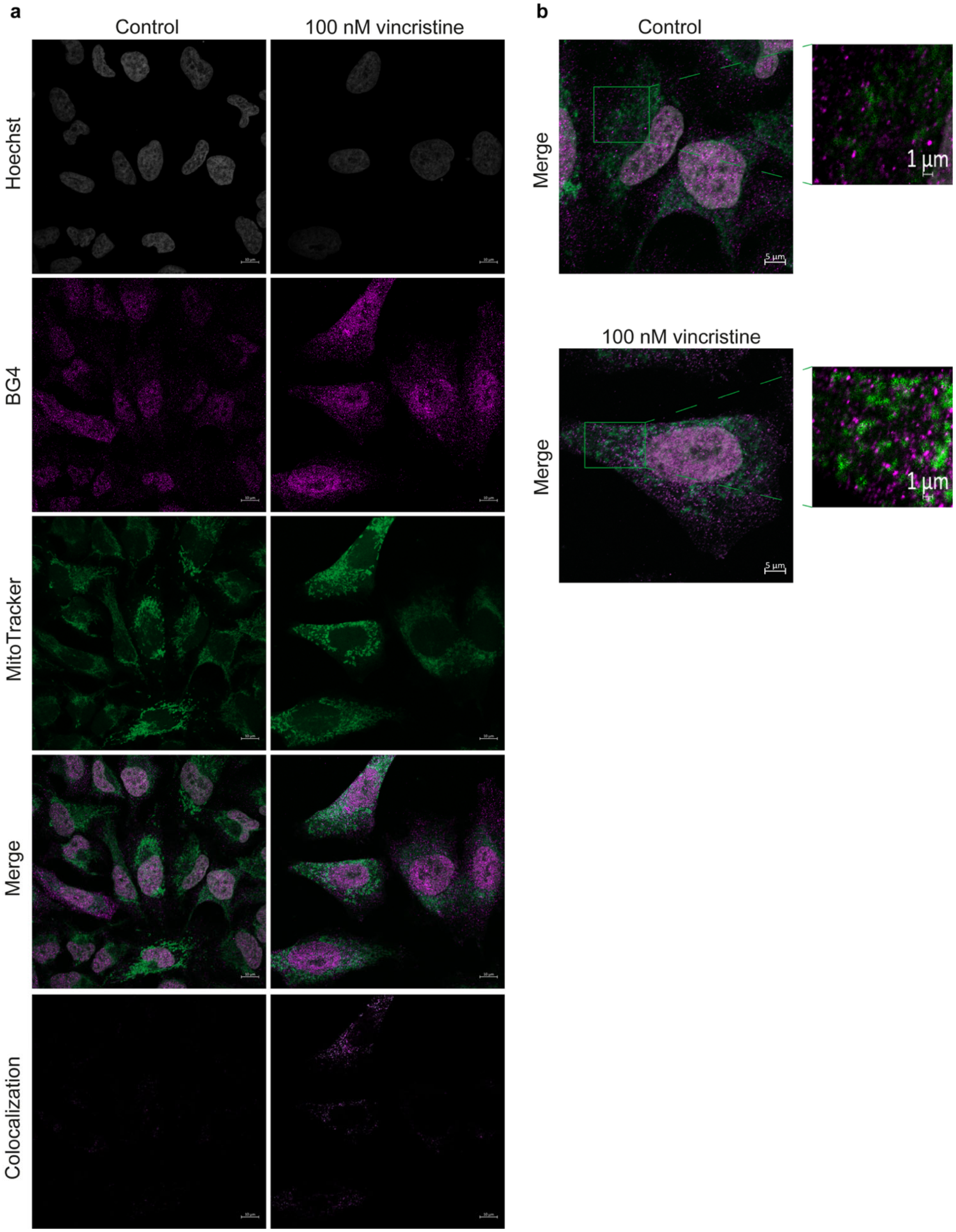

Figure S5. (**a**) Representative immunofluorescence images showing G4 foci formation in U2OS cells following a 24-hour treatment with either 0.5 % DMSO (control) or 100 nM vincristine. Nuclei were stained with the Hoechst solution (grey), G4 structures with BG4 (magenta), and mitochondria with MitoTracker (green). Merged channels and colocalization areas are also reported. Scale bar: 10 µm. (**b**) Colocalization details (scale bar: 5 µm for lower magnification, 1 µm for higher magnification).

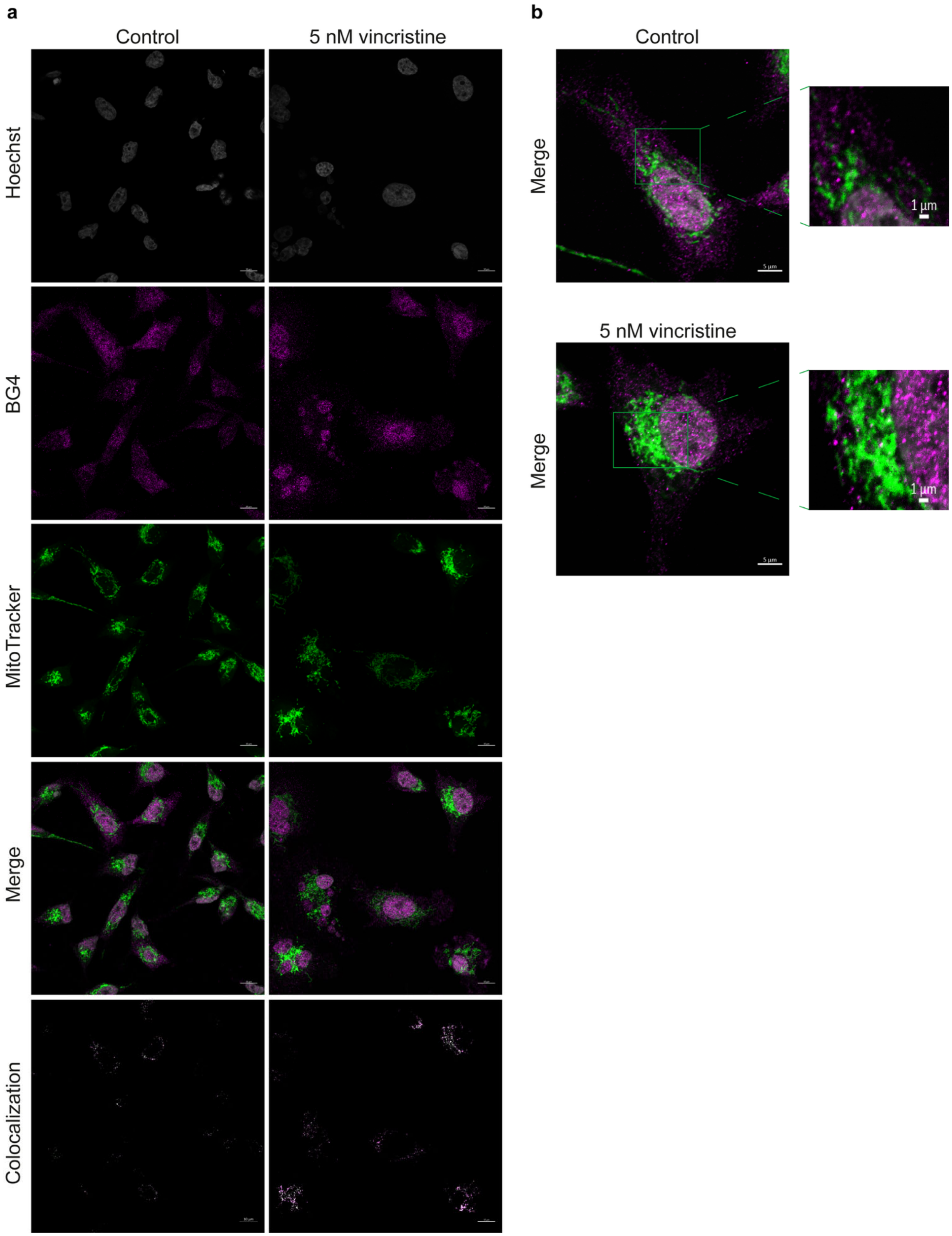

Figure S6. (**a**) Representative immunofluorescence images showing G4 foci formation in MDA-MB-231 cells following a 24-hour treatment with either 0.5% DMSO (control) or 5 nM vincristine. Nuclei were stained with the Hoechst solution (grey), G4 structures with BG4 (magenta), and mitochondria with MitoTracker (green). Merged channels and colocalization areas are also reported. Scale bar: 10 µm. (**b**) Colocalization details (scale bar: 5 µm for lower magnification, 1 µm for higher magnification).

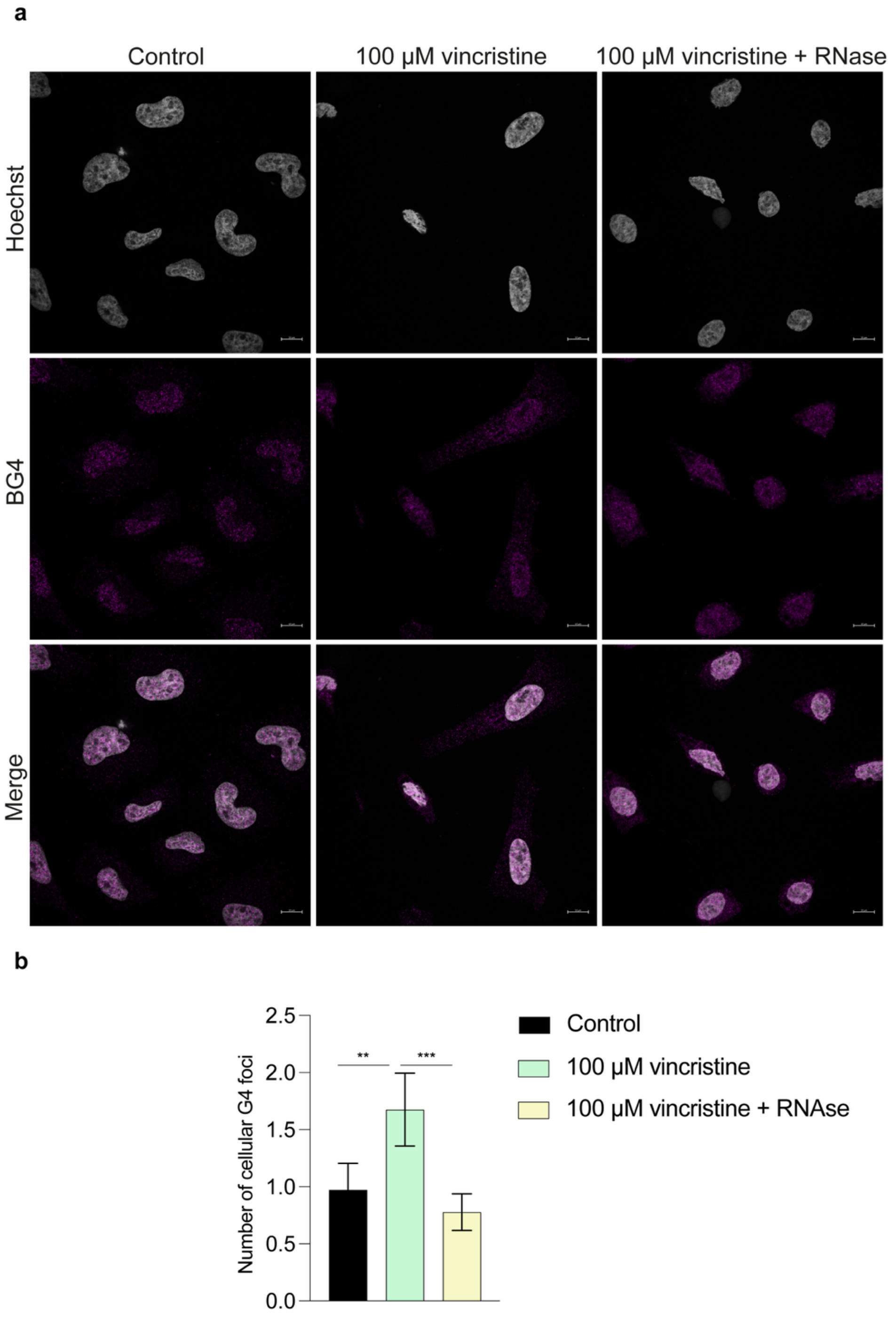

Figure S7. (a) Representative immunofluorescence images showing G4 foci formation in HeLa cells following a 24-hour treatment with either 0.5 % DMSO (control) or 100 μM vincristine. When denoted, pre-treatment with RNase A was used before BG4 staining. Nuclei were stained with the Hoechst solution (grey) and G4 structures with BG4 (magenta). Merged channels are also reported. Scale bar: 10 μm. (b) Quantitative analysis of cellular G-quadruplex foci. Results are expressed as fold change over DMSO-treated control and represent the mean ± SD of two independent experiments. Approximately 100 cells were screened per condition. The statistical significance was calculated using a one-way ANOVA test on GraphPad Prism 10.2.1 (**: $p < 0.01$; ***: $p < 0.001$).

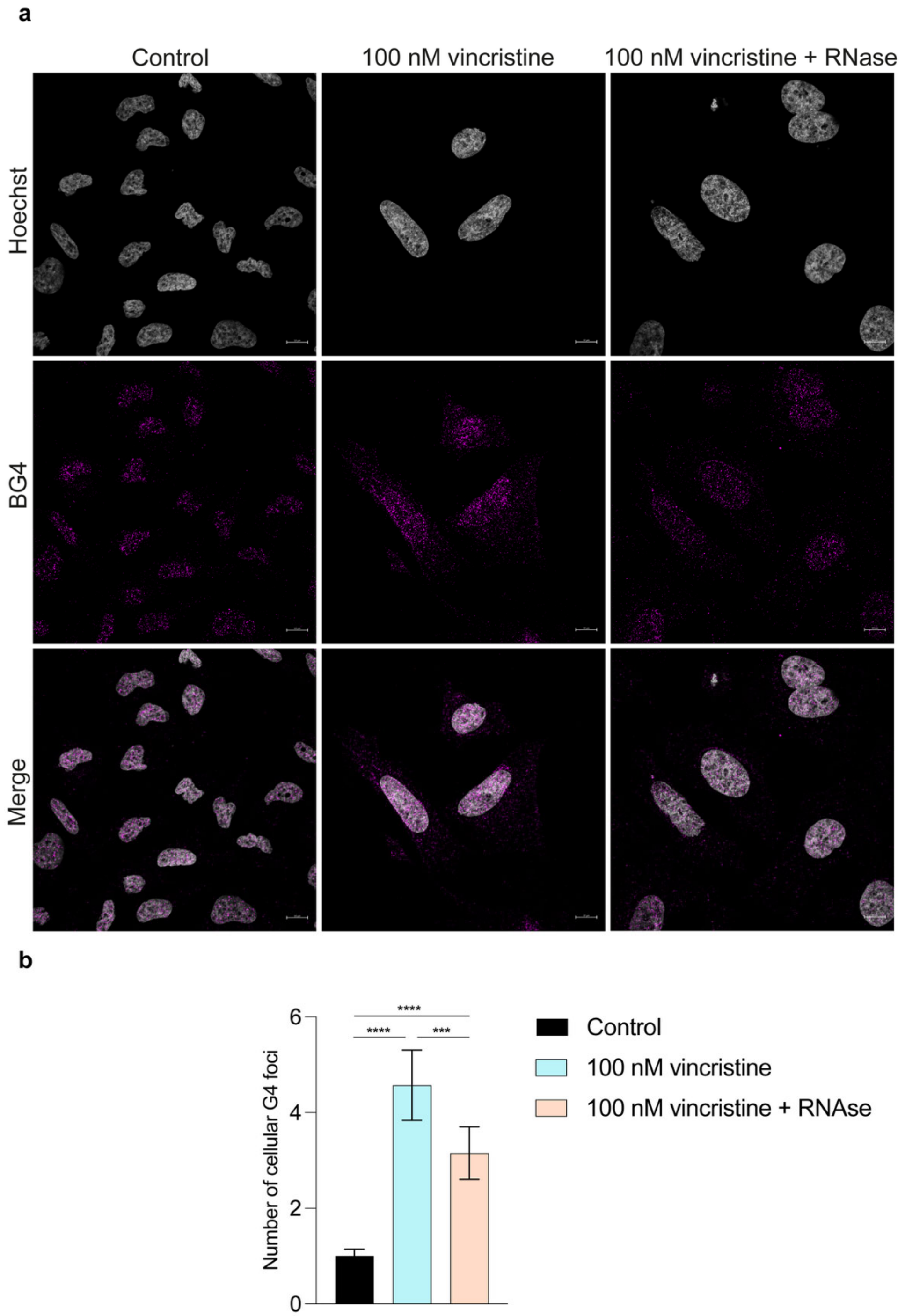

Figure S8. (a) Representative immunofluorescence images showing G4 foci formation in U2OS cells following a 24-hour treatment with either 0.5 % DMSO (control) or 100 nM vincristine. When denoted, pre-treatment RNase A was used before BG4 staining. Nuclei were stained with the Hoechst solution (grey) and G4 structures with BG4 (magenta). Merged channels are also reported. Scale bar: 10 μm. (b) Quantitative analysis of cellular G-quadruplex foci. Results are expressed as fold change over DMSO-treated control and represent the mean ± SD of two independent experiments. Approximately 100 cells were screened per condition. The statistical significance was calculated using a one-way ANOVA test on GraphPad Prism 10.2.1 (***: $p < 0.001$; ****: $p < 0.0001$).

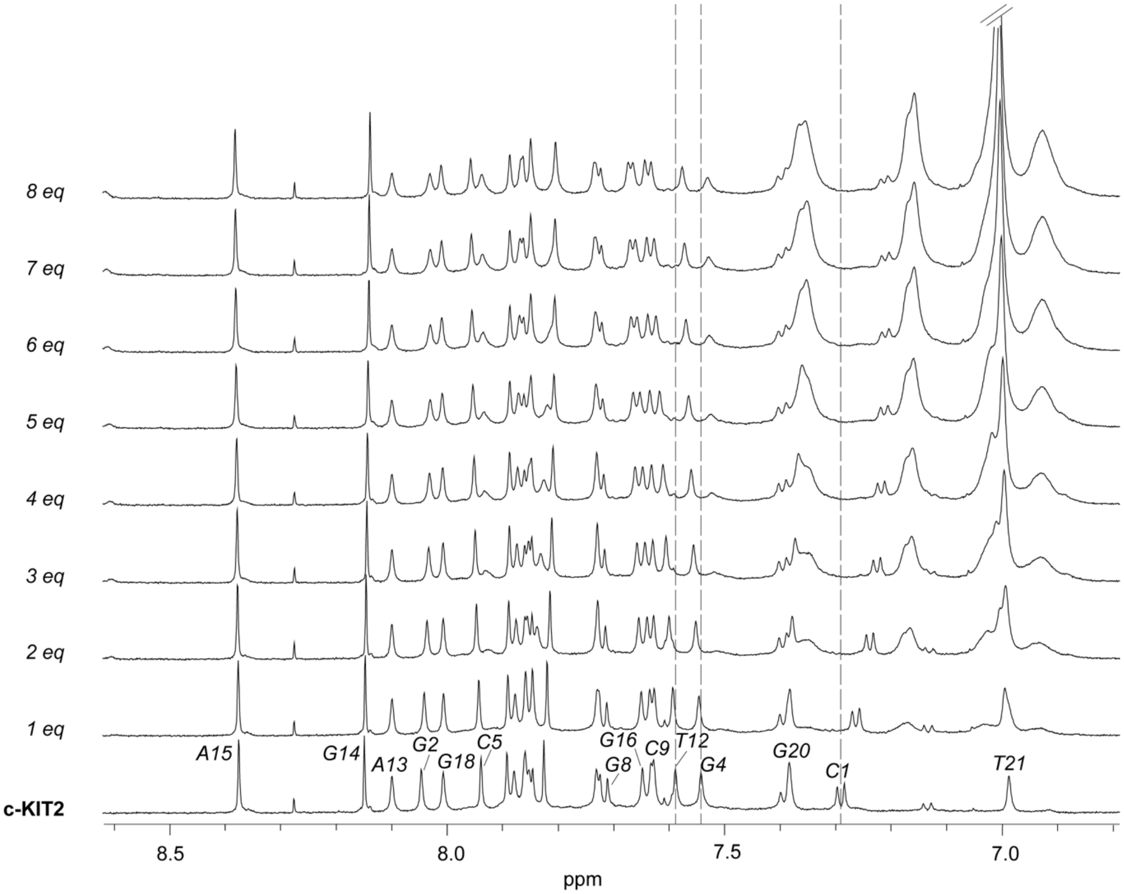

Figure S9. Aromatic region of the ^{1}H-NMR titration spectra for c-*KIT2* G4 upon incremental addition of up to 8 molar equivalents of vincristine at 25 °C.

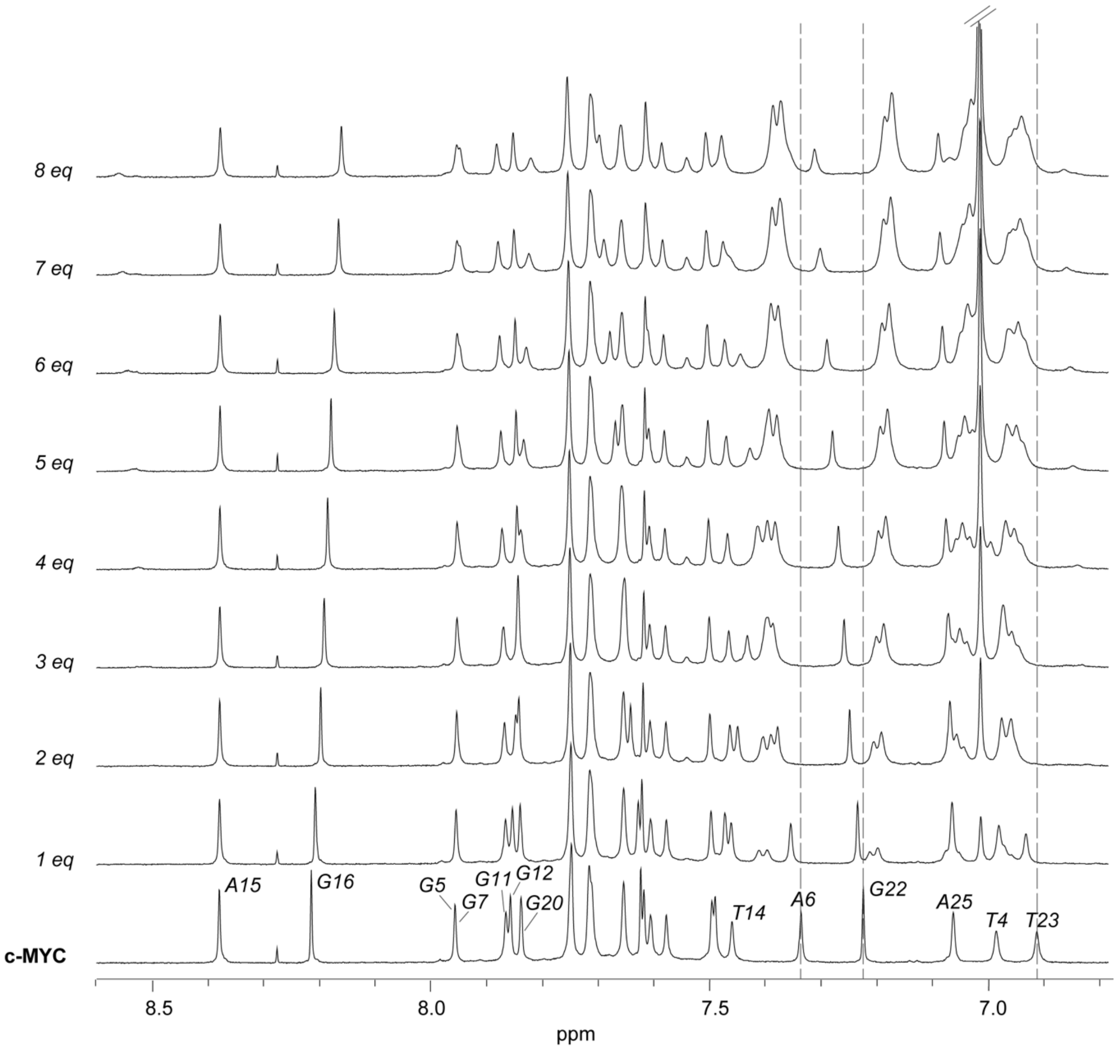

Figure S10. Aromatic region of the ^{1}H-NMR titration spectra for c-*MYC* G4 upon incremental addition of up to 8 molar equivalents of vincristine at 25 °C.

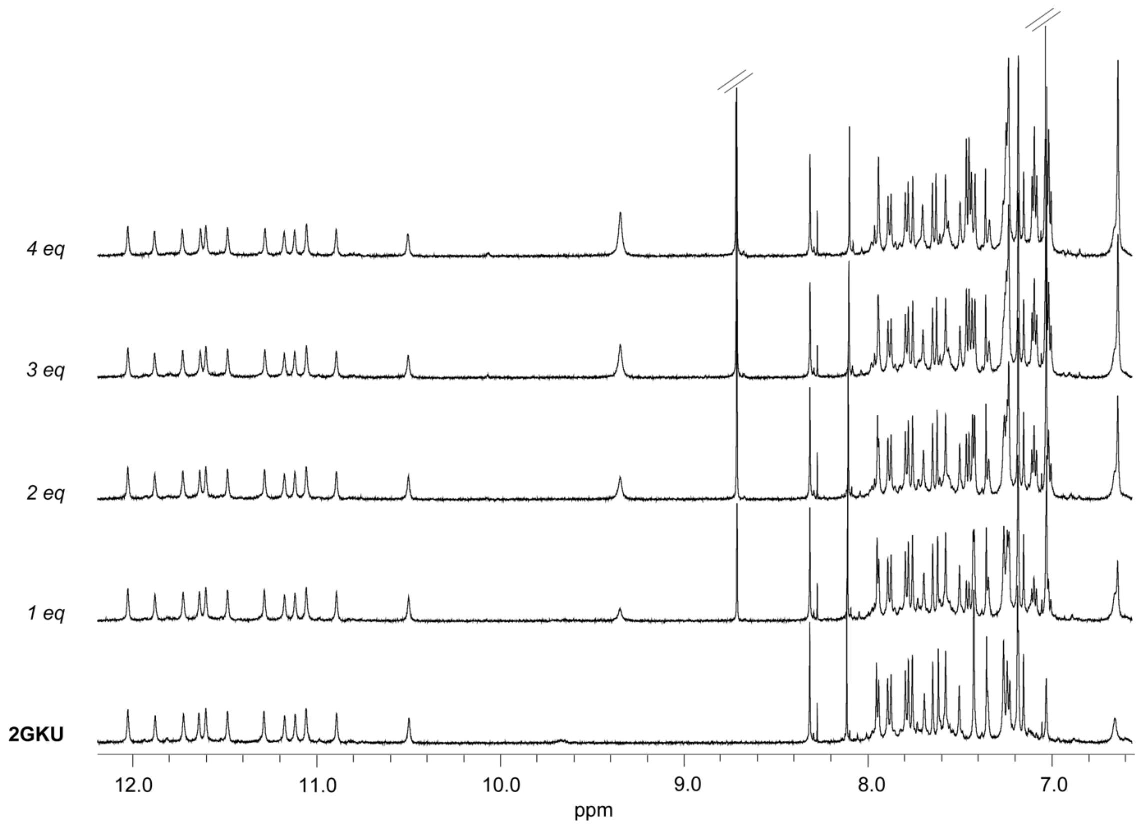

Figure S11. ^{1}H-NMR titration of mutTel24 (PDB: 2GKU) performed by adding up to 4 molar equivalents of vincristine at 25 °C.

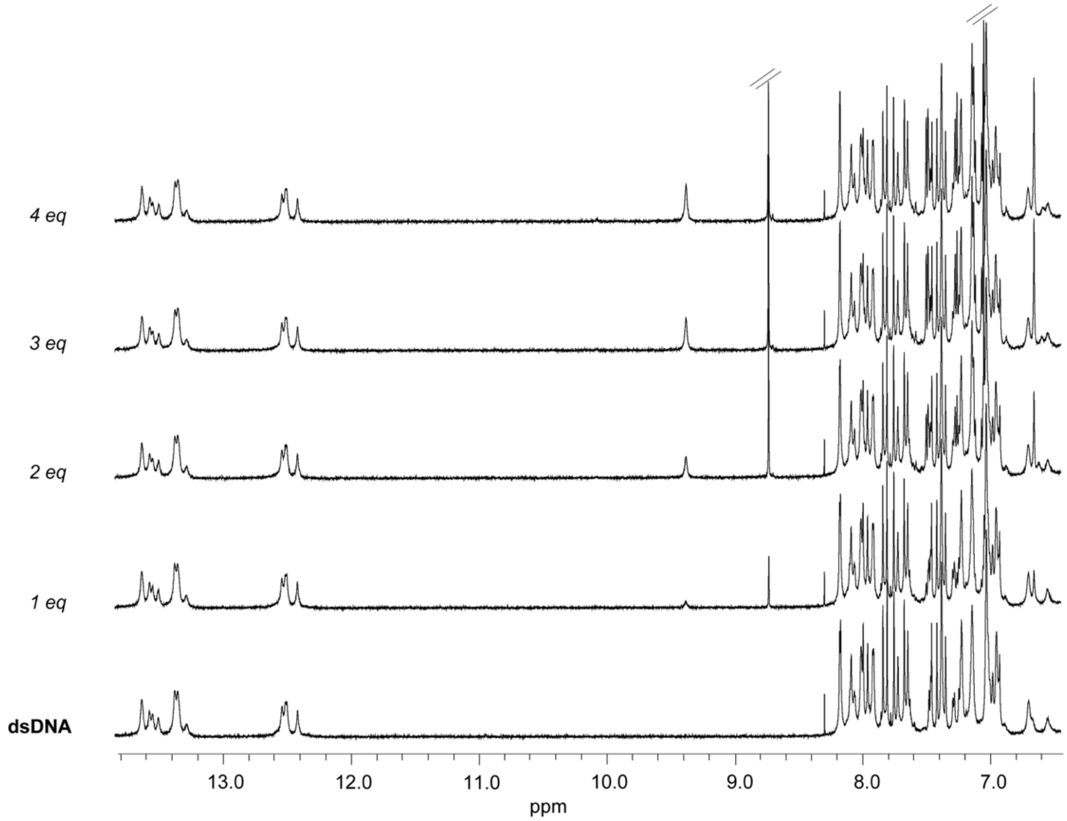

Figure S12. ^{1}H-NMR titration of dsDNA performed by adding up to 4 molar equivalents of vincristine at 25 °C.

a)

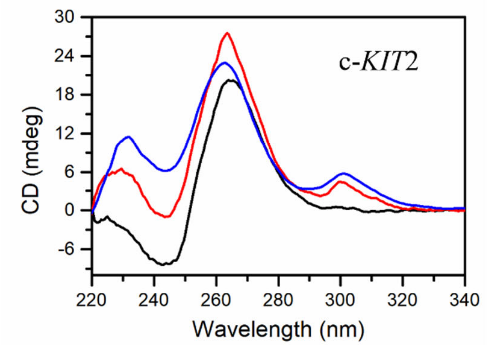

b)

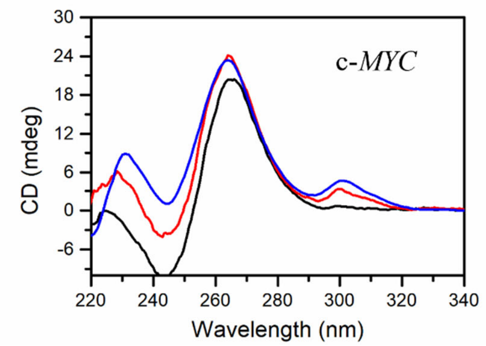

Figure S13. CD spectra at 20 °C of a) c-*KIT2* and b) c-*MYC* in the absence (black line) and presence of 4 (blue line) and 8 (red line) molar equivalents of vincristine.

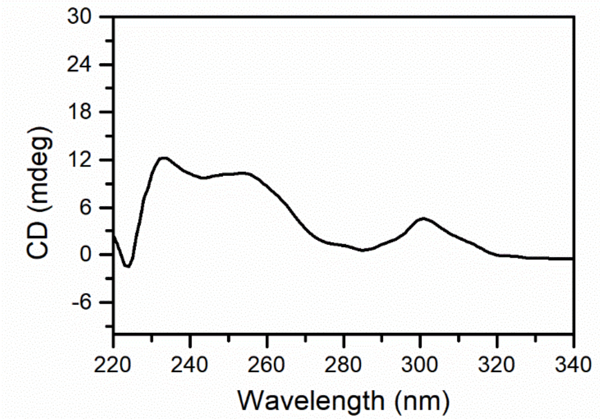

Figure S14. CD spectrum of vincristine recorded at 20 °C, at a concentration of 160 μM (corresponding to the 8 molar equivalents) in an aqueous buffer solution containing 10 mM KH_2PO_4 and 20 mM KCl.

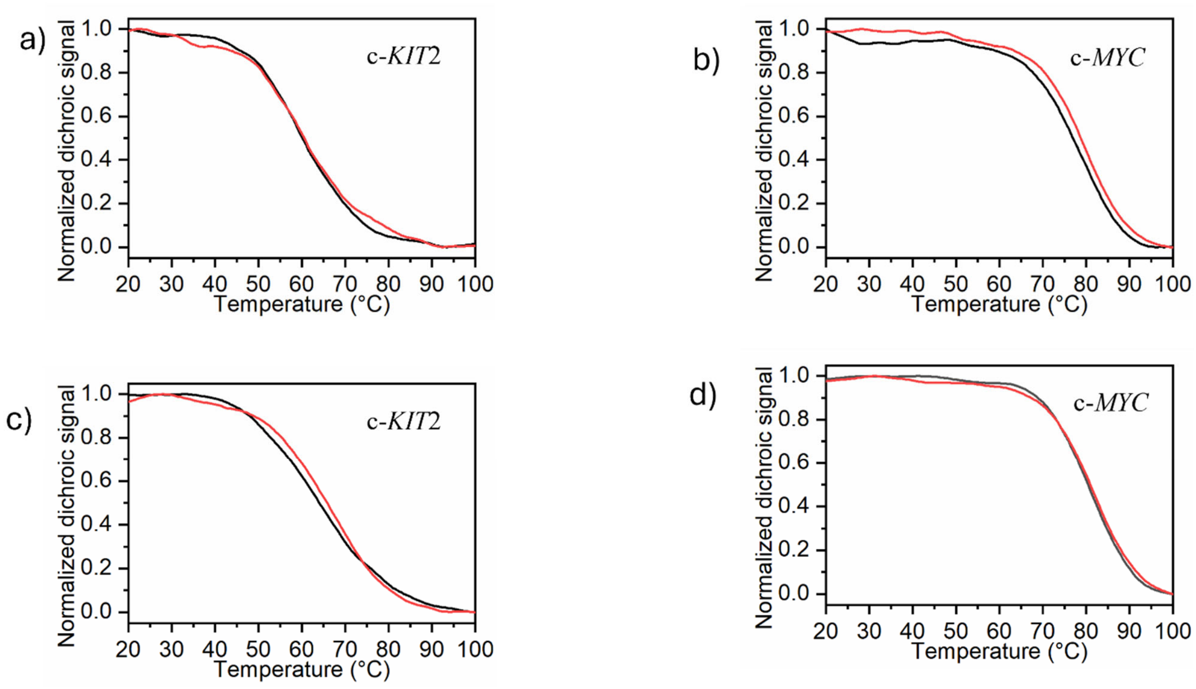

Figure S15. Normalized CD melting curves of c-*KIT2* G4 (left) and c-*MYC* G4 (right) in the absence (black lines) and presence 4 (top) and 8 (bottom) molar equivalents of vincristine.

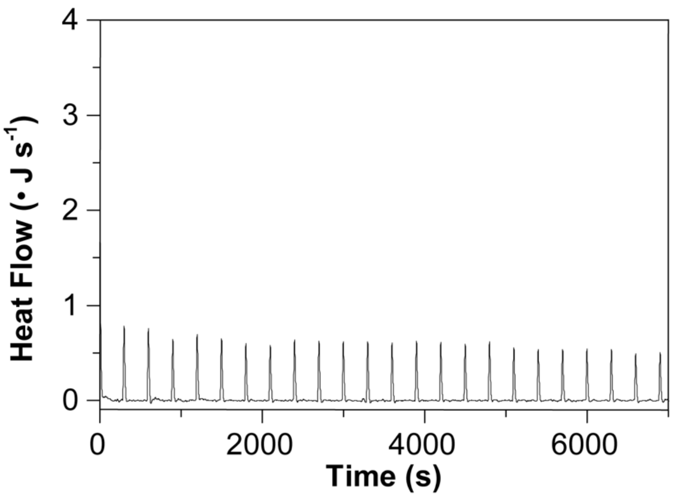

Figure S16. Calorimetric peaks from the isothermal titration of DNA-free buffer with vincristine (amount corresponding to 8 molar equivalents used for the G4s) at 25 °C.

Table S1. Chemical shift perturbations (Δδ) observed in the imino and aromatic proton resonances of the c-*KIT2* G-quadruplex upon titration with 8 molar equivalents of vincristine, recorded at 25 °C at 600 MHz. The shifts (in Hz) were measured relative to the unbound DNA spectrum. Proton assignments are grouped by structural localization (5′ end, central core, 3′ end, and loops). Chemical shift perturbations (Δδ) higher than 20 Hz are shown in bold.

Base	Proton Type	Structural Region	Δδ (Hz)
G14	Imino	5′ end	16.00
G2	**Imino**	**5′ end**	**20.15**
G18	Imino	5′ end	9.56
G6	**Imino**	**5′ end**	**50.72**
G7	Imino	Central	18.23
G19	Imino	Central	11.98
G15	Imino	Central	14.41
G3	Imino	Central	3.68
G8	**Imino**	**3′ end**	**21.97**
G20	Imino	3′ end	3.37
G4	Imino	3′ end	8.18
G16	**Imino**	**3′ end**	**32.03**
G14	Aromatic	5′ end	6.21
G2	Aromatic	5′ end	9.40
G18	Aromatic	5′ end	2.28
C1	**Aromatic**	**5′ end**	**46.62**
G4	**Aromatic**	**3′ end**	**20.63**
T21	Aromatic	3′ end	4.66
A17	Aromatic	Loop	3.83
A13	Aromatic	Loop	0.00
C5	Aromatic	Loop	11.37
T12	**Aromatic**	**Loop**	**25.98**

Table S2. Chemical shift perturbations (Δδ) observed in the imino and aromatic proton resonances of the c-*MYC* G-quadruplex upon titration with 8 molar equivalents of vincristine, recorded at 25 °C at 600 MHz. The shifts (in Hz) were measured relative to the unbound DNA spectrum. Proton assignments are grouped by structural localization (5′ end, central core, 3′ end, and loops). Chemical shift perturbations (Δδ) higher than 20 Hz are shown in bold.

**Base**	**Proton Type**	**Structural Region**	**Δδ (Hz)**
G16	Imino	5′ end	18.72
G7	Imino	5′ end	13.78
G11	**Imino**	**5′ end**	**26.28**
G20	**Imino**	**5′ end**	**26.88**
G8	Imino	Central	19.74
G17	Imino	Central	14.01
G21	Imino	Central	12.81
G12	Imino	Central	9.45
G9	**Imino**	**3′ end**	**71.03**
G13	**Imino**	**3′ end**	**32.09**
G18	Imino	3′ end	2.94
G22	Imino	3′ end	16.01
G16	**Aromatic**	**5′ end**	**32.22**
G7	Aromatic	5′ end	5.15
G11	Aromatic	5′ end	9.28
G20	Aromatic	5′ end	8.82
G5	Aromatic	5′ end	2.91
T4	Aromatic	5′ end	11.77
A6	**Aromatic**	**5′ end**	**24.8**
G22	**Aromatic**	**3′ end**	**53.17**
T23	**Aromatic**	**3′ end**	**28.04**
A25	Aromatic	3′ end	16.68
G12	**Aromatic**	**Central**	**22.50**
A15	Aromatic	Loop	0.98
T14	Aromatic	Loop	11.19
